# Comparative effectiveness and tolerance of immunosuppressive treatments for idiopathic membranous nephropathy: A network meta-analysis

**DOI:** 10.1371/journal.pone.0184398

**Published:** 2017-09-12

**Authors:** Song Ren, Ying Wang, Li Xian, Tadashi Toyama, Meg Jardine, Guisen Li, Vlado Perkovic, Daqing Hong

**Affiliations:** 1 Renal Division and Institute of Nephrology, Sichuan Academy of Medical Science & Sichuan Provincial People’s Hospital, School of Medicine, University of Electronic Science and Technology of China, Chengdu, China; 2 The George Institute for Global Health, University of Sydney, Sydney, Australia; 3 Division of Nephrology, Kanazawa University Hospital, Kanazawa city, Japan; 4 Concord Repatriation General Hospital, Concord, Australia; Istituto Di Ricerche Farmacologiche Mario Negri, ITALY

## Abstract

**Background:**

Immunosuppressive agents in general are shown to prevent renal progression and all-cause mortality in idiopathic membranous nephropathy (IMN) patients with nephrotic syndrome. However, the efficacy and safety of different immunosuppressive treatments have not been systematic assessed and compared. A network meta-analysis was performed to compare different immunosuppressive treatment in IMN.

**Methods:**

Cochrane library, MEDLINE, EMBASE and trial register system were searched for randomized controlled trials reporting the treatments for IMN to May 3, 2016. Composite endpoint of mortality or end-stage kidney disease (ESKD), complete or partial proteinuria remission and withdrawal because of treatment adverse events were compared combing direct and indirect comparison using network meta-analysis. Ranking different immunosuppressive treatments in the outcomes were analyzed by using surface under the cumulative ranking curve (SUCRA).

**Results:**

Total 36 randomized controlled trials (n = 2018) covering 11 kinds of treatments were included. Compared with non-immunosuppressive treatment, only cyclophosphamide (CTX) and chlorambucil significantly reduced the risk of composite outcome of mortality or ESKD while combining the direct and indirect comparison (OR = 0.31, 95%CI: 0.12–0.81 and OR = 0.33, 95%CI: 0.12–0.92). CTX increased the composite outcome of complete remission (CR) or partial remission (PR) (OR = 4.29, 95%CI: 2.30–8.00) but chlorambucil did not (OR = 1.58, 95%CI: 0.80–3.12) as compared with non-immunosuppressive treatment. Chlorambucil also significantly increased the withdrawal risk (OR = 3.34, 95%CI: 1.37–8.17) as compared to CTX. Both tacrolimus (OR = 3.10, 95%CI: 1.36–7.09) and cyclosporine (CsA) (OR = 2.81, 95%CI: 1.08–7.32) also significantly increased the rate of CR or PR as compared with non-immunosuppressive treatment (without significant difference as compared with CTX), while ranking results showed that cyclosporine or tacrolimus was with less possibility of drug withdrawal as compared to CTX.

**Conclusions:**

Cyclophosphamide and chlorambucil reduce risk of ESKD or death in IMN with nephrotic range proteinuria, but carry substantial toxicity that may be lower for cyclophosphamide. Tacrolimus and cyclosporine increase the possibility of proteinuria remission with less drug withdrawal, but the effects on kidney failure remain uncertain.

## Introduction

Idiopathic membranous nephropathy (IMN) is one of the commonest causes of primary nephrotic syndrome in adults [1]. Approximately one-third of affected individuals will have a complete and spontaneous remission of proteinuria, while another third develop persistent proteinuria with long-term preservation of renal function. However, the remaining third of patients will progress to end-stage kidney disease (ESKD), and the risk is higher in people with nephrotic range proteinuria [2,3].

Overall, immunosuppressive therapy has been suggested to reduced proteinuria, all-cause mortality and progression to ESKD [4]. The Kidney Disease Improving Global Outcomes (KDIGO) guidelines recommend steroids plus alkylating agents in patients at high risk of developing ESKD based on severe proteinuria. People with contraindications for alkylating agents or who do not tolerant them, are recommended to receive steroids plus calcineurin inhibitors, (such as tacrolimus and cyclosporine) as second line therapy[5]. However, the quality of the evidence has been noted to be moderate leaving residual uncertainty about the optimal treatment approach, and a number of additional randomized-controlled trials (RCTs) evaluating novel immunosuppressive therapies for IMN have been published subsequent to these guidelines.

In this study, we used network meta-analysis to directly and indirectly compare the effects of different immunosuppressive regimens on renal survival, proteinuria remission and tolerability in people with IMN and nephrotic range proteinuria.

## Materials and methods

### Data sources and searches

The Cochrane Central Register of Controlled Trials (CENTRAL), MEDLINE and EMBASE databases were systematically searched for randomized controlled trials in people with IMN up to May 3, 2016. The search terms used consisted of IMN and randomized controlled trials (**S1 Table**). We searched two additional websites [www.controlled-trials.com, and www.clinicaltrials.gov] for ongoing clinical trials, and manually checked the reference lists of included studies to identify additional eligible studies.

### Study selection

Studies of immunosuppressive treatments in IMN were included. We included studies of people with biopsy proven IMN and nephrotic range proteinuria (urinary protein excretion >3.5 g/24h). Membranous nephropathy secondary to autoimmune diseases, cancer, infections (including B and C hepatitis) and drugs, or atypical membranous nephropathy were excluded from our review.

Type of interventions: We included any treatment expected to primarily act via an immunosuppressive effect. Agents without clear mechanism of immunosuppressive effects (such as traditional Chinese medicine) were excluded.

Comparators: Different immunosuppressive treatments were compared with each other or with non-immunosuppressive treatment (placebo, renin angiotensin system blockers or other supportive therapies). We excluded the trials with more than two immunosuppressive agents in one arm (except steroids) due to difficulty in differentiating the treatment effects as a result of the combined effect or the sum of the monotherapy effect. Studies that assessed the same immunosuppressive agents with different doses in different arms but no other comparator were excluded. Trials with a follow-up of less than 6 months were excluded, as were cross-over trials. The abstracts were initially screened by two researchers (SR and LX). Disagreements were resolved through discussions (SR, LX, DH and GL).

### Data extraction and quality assessment

Data extraction was undertaken independently by 2 investigators (SR and LX) using a standardized electronic form. Disagreements in abstracted data were resolved by a third investigator (DH). The abstracted data included baseline characteristics of the participants such as age, sex, serum creatinine, serum albumin and proteinuria value. The main outcomes included 1) the composite of all-cause mortality or ESKD (defined as start of dialysis or renal transplantation); 2) complete or partial remission (defined by the authors in each study); 3) withdrawal due to the drug related toxicity. Other outcomes included adverse reactions such as infection, bone marrow suppression, abnormal liver function, incidence of diabetes mellitus or hypertension, or as defined by the study authors. Study quality was judged by the Jadad score[6] and Cochrane Collaboration tool[7] for assessing the risk of bias. We considered the study of low-quality if the Jadad score was less than 3, or higher-quality if the Jadad score was more than or equal to 3. The risk of bias assessment included random sequence generation, allocation concealment, blinding of participants and personnel, blinding of outcome assessment, incomplete outcome data, selective reporting and other bias[8]. We summarized both individual and aggregate risk of bias data for the included studies.

### Data synthesis and statistical analysis

We use STATA (version 14.0) to perform network meta-analysis with a random-effects mixed-treatment comparisons model for multi-armed trials within the frequency probability method on the effects of main outcomes. Odds ratios (OR) and their 95% confidence intervals (CI) were used to compare treatment effects for each dichotomous outcome. To summarize the effectiveness and tolerance of all treatments, we also calculated the surface under the cumulative ranking curve (SUCRA).

The heterogeneity of the included studies was large, and it is unavoidable. So we perform a consistency test to differences between direct and various indirect comparisons by the node-splitting approach for the main outcomes. The *p* value was calculated by the node-splitting method, which separated evidence on a particular comparison into direct and indirect evidence[9]. We used consistency model to do network meta-analysis when there was no statistically significant difference between direct and indirect comparison.

For the main outcomes, we also performed sensitivity analysis according to study quality (excluding studies with Jadad scores less than 3). As the participants’ follow-up time may influence effects on mortality or ESKD, we also performed a sensitivity analysis to exclude studies with follow-up time of less than two years.

## Results

### Description of the included studies

**Fig 1:**
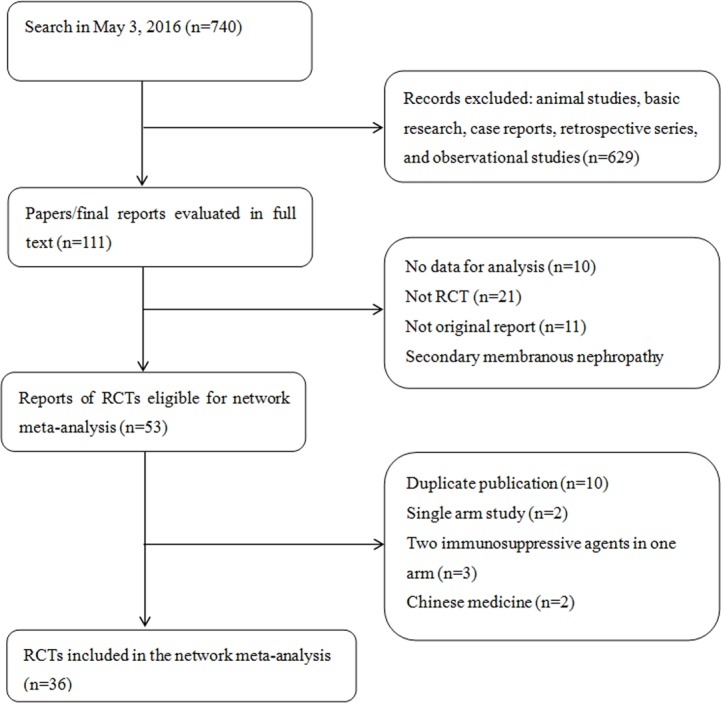
Study selection flow chart. RCT, randomized controlled trial.

The search retrieved 750 citations for screening, from which 36 studies [10–45] published from 1974 to 2016 involving 2018 participants and covering 11 kinds of treatments were eventually included (**Fig 1**). All studies were published in English except one in Japanese literature[42]. Four studies were three-armed trials [11,13,35,43] and the others had two arms. Five studies were only published in abstract form [24,34–36,45]. Eighteen studies used ACEI/ARB or placebo as the control group and immunosuppressive agents as the treatment group (**S2 Table**).

**Fig 2:**
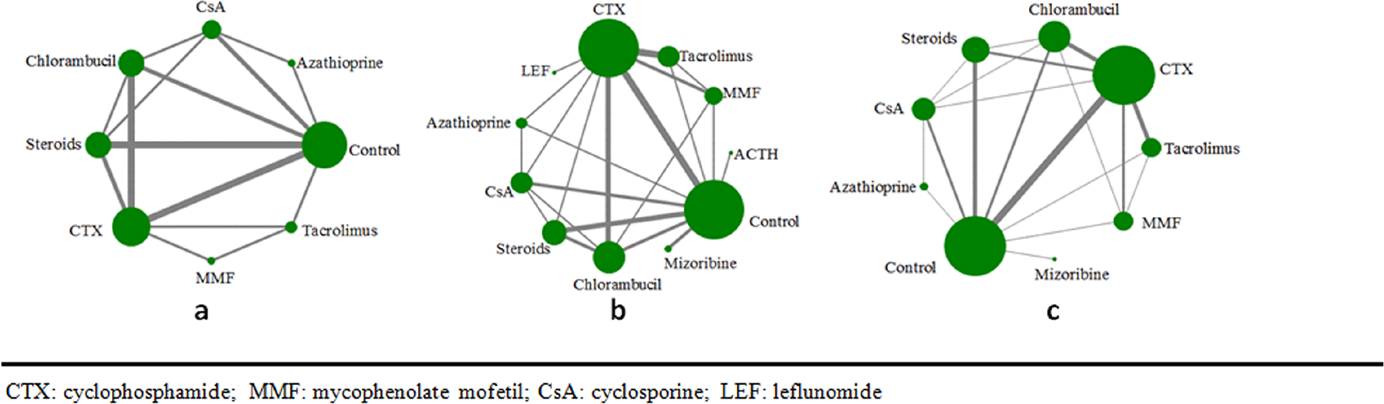
a. Network of eligible comparisons for total mortality or ESKD. The width of lines is proportional to the number of studies compared in every pair of treatments, and the size of nodes is proportional to the total sample size of each treatment. b. Network of eligible comparisons for the outcome of total remission. c. Network of eligible comparisons for the outcome of treatment withdrawal.

Overall, 19 RCTs reported effects on all-cause mortality or ESKD, 31 RCTs reported effects on complete or partial remission, and 21 RCTs reported effects on treatment intolerance. The average follow-up time was 33months (9-120months). Trials generally had a small sample size with a median of 56 (9–158) patients (**Fig 2**).

Seventeen of 36 studies had a Jadad score of less than 3. The risk of bias of each study was assessed using Review Manager 5.3 and summarized in the **S1**and **S2 Figs**.

### Composite endpoint of mortality or ESKD

Effects on all-cause mortality or ESKD were reported in 19 studies including 8 types of immunosuppressive therapies. Among these, 14 studies reported effects on all-cause mortality alone, including 7 types of immunosuppressive therapies, and 16 studies separately reported effects on ESKD, involving 6 types of immunosuppressive therapies. There was no endpoint events reported in trials involving mizoribine, ACTH or leflunomide.

The results of the network analysis of eligible comparisons for total mortality or ESKD are shown in Fig 2A. There was no statistically significant difference between the direct and indirect comparisons using the node-splitting method (*p* > 0.05). We therefore used the consistency model to undertake network meta-analysis.

**Fig 3:**
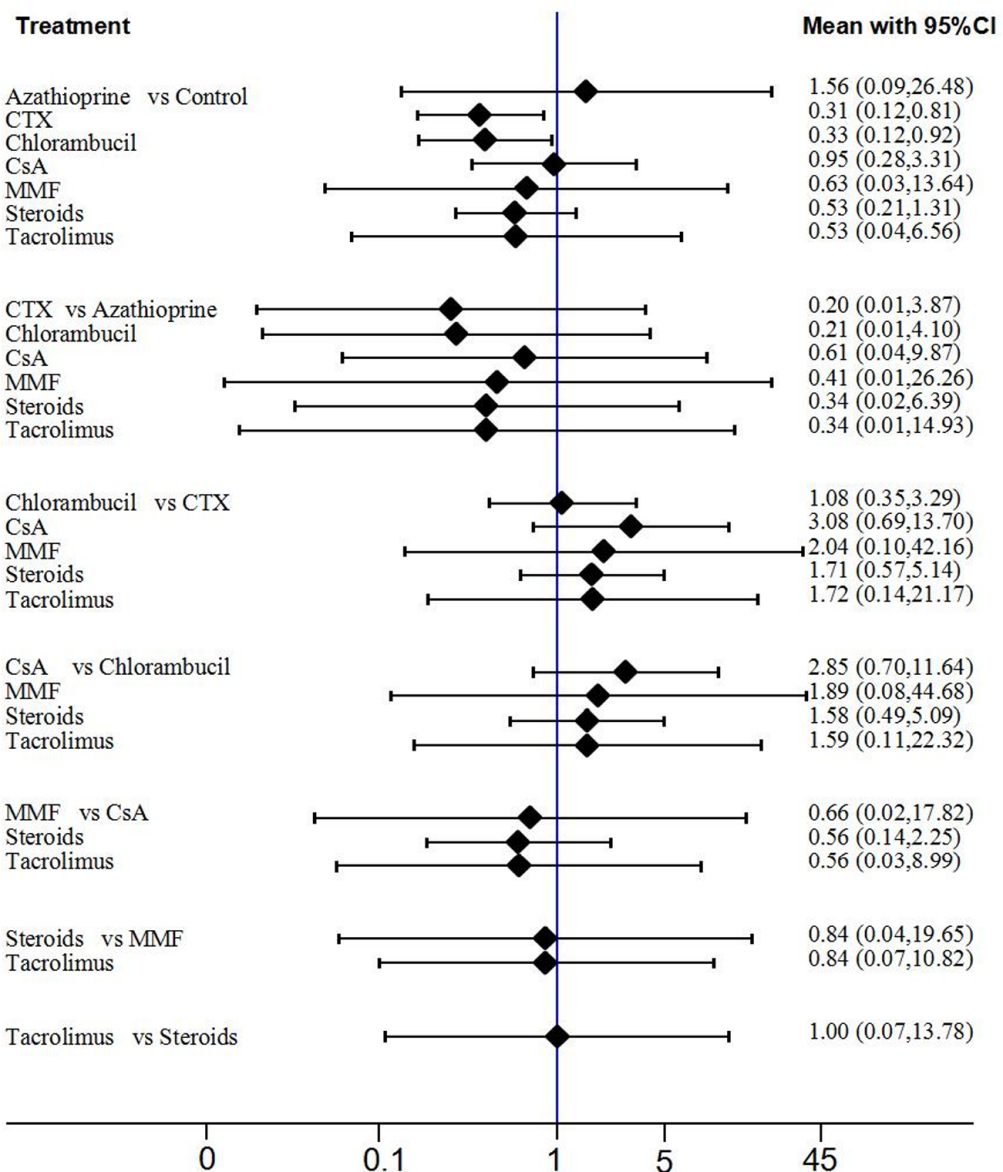
The results of network meta-analysis (total mortality or ESKD). All treatments were compared with each other. In the first group, 7 treatments were compared with the control group. In the second group, 6 treatments were compared with the azathioprine group. In the third group, 5 treatments were compared with the CTX group. In the fourth group, 4 treatments were compared with the chlorambucil group. In the fifth group, 3 treatments were compared with the CsA group. In the sixth group, 2 treatments were compared with the MMF group. In the last group, tacrolimus compared with the steroids group.

The findings of the network meta-analysis for mortality or ESKD is shown in **Fig 3**(direct and all comparison results). Ranking of treatments is presented in **S3 Fig.** Compared with non-immunosuppressive treatment, only cyclophosphamide (CTX) and chlorambucil significantly reduced the risk of the composite outcome of mortality or ESKD when combining the direct and indirect comparison (OR = 0.31, 95%CI 0.12–0.81 and OR = 0.33, 95%CI 0.12–0.92, respectively). No clear effects were able to be identified with the other immunosuppressive agents assessed.

Fifteen high-quality studies including 7 types of immunosuppressive therapies were included to perform further sensitivity analysis and found similar results (**S6 Fig**) Exclusion of studies with follow up period of <2 years also did not change the primary conclusions, although confidence intervals were widened.

### Complete or partial remission

**Fig 4:**
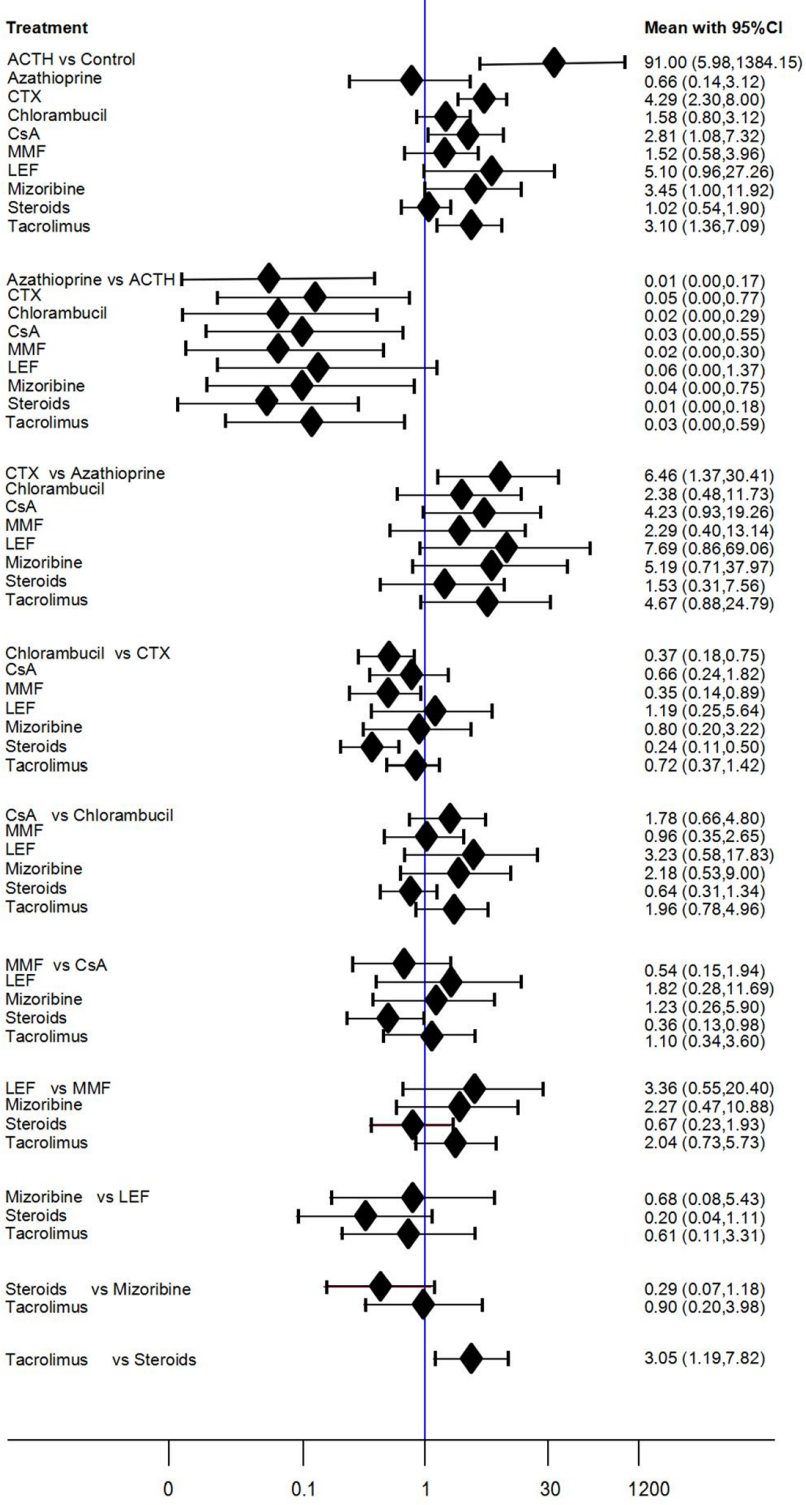
The results of network meta-analysis (complete or partial remission). In the first group, 10 treatments were compared with the control group. In the second group, 9 treatments were compared with the ACTH group. In the third group, 8 treatments were compared with the azathioprine group. In the fourth group, 7 treatments were compared with the CTX group. In the fifth group, 6 treatments were compared with the chlorambucil group. In the sixth group, 5 treatments were compared with the CsA group. In the seventh group, 4 treatments were compared with the MMF group. In the eighth group, 3 treatments were compared with the LEF group. In the ninth group, 2 treatments were compared with the mizoribine group and in the last group, tacrolimus compared with the steroids group.

A total of 31 RCTs including 11 immunosuppressive therapies reported effects on complete or partial remission, including cyclophosphamide (15 studies), control (18 studies), chlorambucil (9 studies), steroids (9 studies), cyclosporine (6 studies), tacrolimus (5 studies), mycophenolate mofetil (4 studies), azathioprine (3 studies), mizoribine (2 studies), ACTH (1 study), and leflunomide (1 study). The network analysis of eligible comparisons for the total remission outcome of the network meta-analysis is shown in **Fig 2**. Direct and indirect estimates by the node-splitting method identified no statistically significant differences. Compared with non-immunosuppressive therapies, four immunosuppressive agents showed significantly improve CR or PR. ACTH showed the best therapeutic effect among all the immunosuppressive therapies (OR = 91.00, 95%CI 5.98–1384.15), followed by CTX (OR = 4.29, 95%CI 2.30–8.00), tacrolimus (OR = 3.10, 95%CI 1.36–7.09), CsA (OR = 2.81, 95%CI 1.08–7.32). Chlorambucil (OR = 1.58, 95%CI 0.8–3.12) and MMF (OR = 1.52, 95%CI 0.58–3.96) didn’t significantly improve CR or PR compared with non-immunosuppressive therapies (**Fig 4)**. The rank probabilities of all treatments calculated using the SUCRA is shown in **S4 Fig**.

A sensitivity analysis that excluded lower quality studies showed that only two immunosuppressive agents (CTX and tacrolimus) significantly improve CR or PR (OR = 3.73, 95%CI 1.85–7.48 and OR = 3.69, 95%CI 1.49–9.14), respectively, compared with non-immunosuppressive therapies (**S7 Fig).**

### Withdrawal of treatments

**Fig 5:**
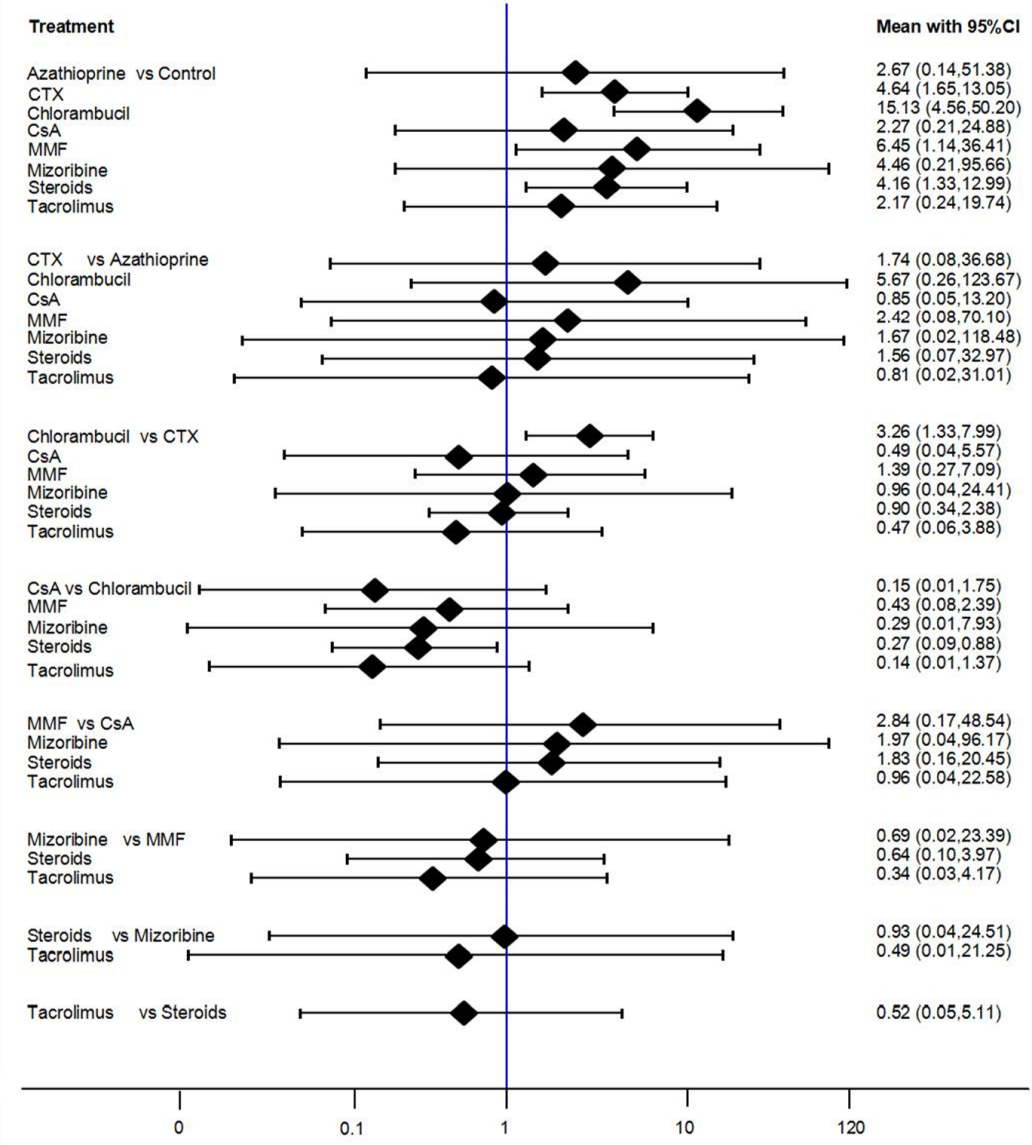
The results of network meta-analysis (withdrawal of treatments). In the first group, 8 treatments were compared with the control group. In the second group, 7 treatments were compared with the azathioprine group. In the third group, 6 treatments were compared with the CTX group. In the fourth group, 5 treatments were compared with the chlorambucil group. In the fifth group, 4 treatments were compared with the CsA group. In the sixth group, 3 treatments were compared with the MMF group. In the seventh group, 2 treatments were compared with the mizoribine group and in the last group, tacrolimus compared with the steroids group.

A total of 21 RCTs assessing 9 immunosuppressive agents reported effects on withdrawal from treatment. The network analysis of eligible comparisons for the total tolerance outcome of the network meta-analysis is shown in **Fig 2**. In the network meta-analysis comparison, the control group had the lowest withdrawal rate and best tolerance. Compared with the control group, chlorambucil was associated with the highest withdrawal rates (OR = 13.73, 95%CI 4.23–44.59), followed by MMF (OR = 6.17, 95%CI 1.10–34.58), mizoribine (OR = 4.46, 95%CI 1.21–91.66), CTX (OR = 4.11, 95%CI 1.52–11.10) and steroids (OR = 3.82, 95%CI 1.24–11.75). Compared with chlorambucil, the withdrawal rates of tacrolimus and CsA were lower (OR = 0.26, 95%CI: 0.06–1.18 and OR = 0.15, 95%CI 0.02–1.21). There were no statistically significant differences between other immunosuppressive agents and the control group. Calcineurin inhibitors showed the best tolerance among all immunosuppressive agents. (**Fig 5**and **S5 Fig)**

After excluding low-quality studies, the sensitivity analysis indicated that ranking of the drug tolerance remained unchanged, with alkylating agents having the highest withdrawal rates (**S8 Fig)**.

### Other adverse reactions associated with use of immunosuppressive agents

Overall, patients treated with immunosuppressive agents were reported to have an increased risk of infection (e.g. pneumonia, cystitis and skin infection), and alkylating agents have a higher risk of myelosuppression (e.g. leukopenia or thrombocytopenia). CTX, CsA and azathioprine was associated with hepatotoxicity, in particularly elevated serum transaminases. The following adverse events were analyzed: infection, bone marrow suppression, and abnormal liver function, incidence of diabetes mellitus (DM) or hypertension.

The occurrence of adverse reactions according to treatments for IMN is listed in **Table 1**. Infection was the most common adverse events associated with various immunosuppressive therapies. The occurrence rate of infection was 7. 9% reported in 21 studies including 160 participants. Three immunosuppressive agents associated with the highest risk of infection were tacrolimus 21.5% (38/177), MMF 19.7% (14/71) and CTX 15.2% (68/448). There were no infections reported with three immunosuppressive agents: mizoribine, ACTH, leflunomide. Myelosuppression was seen with 4 immunosuppressive agents (CTX, chlorambucil, MMF and azathioprine). Among them, azathioprine (14.71%) was associated with highest incidence rates followed by MMF (8.45%), chlorambucil (7.79%) and CTX (2.23%). Deranged liver function tests were seen in patients treated with tacrolimus or CTX. Diabetes mellitus or glucose intolerance was reported in 30 patients (16.9%) treated with tacrolimus. 122 patients relapse after remission of proteinuria (6.0%), patients who are treated with CsA are more likely to develop relapse (21.2%), followed by chlorambucil (16.4%), azathioprine (14.7%). Overall, tacrolimus, azathioprine and MMF were more commonly associated with adverse reactions with the adverse events rates of 49%, 34%, and 32%, respectively.

**Table 1:** The adverse reaction of 11 kinds of treatments for IMN.

Treatments (N)	Infection	Bone marrow suppression	Abnormal liver function	Incidence of hypertension	Incidence of DM	Relapse
CTX (n = 448)	68	10	18	2	19	4
Control (n = 497)	6	0	0	1	2	8
Chlorambucil (n = 244)	19	19	1	1	1	40
Tacrolimus (n = 177)	38	0	10	8	30	2
CsA (n = 137)	8	0	0	12	0	29
MMF (n = 71)	14	6	2	1	1	2
Steroids (n = 309)	5	0	0	6	1	32
Azathioprine (n = 34)	2	5	1	3	0	5
Mizoribine (n = 62)	0	0	0	0	0	0
ACTH (n = 15)	0	0	0	0	0	0
LEF (n = 24)	0	0	0	0	0	0

CTX: cyclophosphamide; CsA: cyclosporine; MMF: mycophenolate mofetil; LEF: leflunomide

## Discussion

Membranous nephropathy is prevalent and could increase the risk of renal function lost and subsequent mortality. In 2009, Beck et al [46] first reported that M-type PLA2 receptor (PLA2R) was a major target antigen for IMN and about 70% of patients with IMN had autoantibodies to PLA2R in their serum. Thrombospondin type 1 domain–containing 7A (THSD7A) was described as a new autoantigen involved in adult idiopathic membranous nephropathy. Tomas et al [47] found 15 of 154 patients with idiopathic membranous nephropathy had circulating autoantibodies to THSD7A but not to PLA2R receptor antibodies. Subsequently, more clinical studies have found THSD7A positivity by testing for circulating antibodies in IMN patients or THSD7A antigen deposition in renal tissue by immunohistochemistry[48–50]. We had evaluated the prevalence of THSD7A in the IMN patients (The article has not yet been published), and the result shows that the estimated prevalence of THSD7A in patients with IMN is 3% and higher prevalence in the PLA2R-negtive patients is 10%. Combined with the detection results of PLA2R and THSD7A can improve the diagnostic level of IMN.

Immunosuppressive agents were used widely in patients with idiopathic membranous nephropathy. In this review, we focused on the outcomes most likely to be important to patients in making decisions, specifically ESKD or death, remission as well as tolerability and side effects. The results indicate that alkylating agents are the only immunosuppressive agents with proven benefits for the composite outcome of ESKD or mortality, and were the immunosuppressive agents most likely to induce remission of proteinuria. The likelihood of complete or partial remission was significantly increased with ACTH, mizoribine, CsA and tacrolimus, as compared with non-immunosuppressive treatment. Conversely, alkylating agents were also associated with a higher risk of withdrawal from the therapy, while calcineurin inhibitors (tacrolimus and CsA) appeared to be well tolerated despite high reported rates of adverse effects with tacrolimus in particular.

Previous studies have demonstrated that glucocorticoids alone had little effective in increasing remission of proteinuria. Therefore, glucocorticoid plus immunosuppressive agents are often used for idiopathic membranous nephropathy. Our findings support the recommendations of the KDIGO guideline where alkylating agents are recommended as the preferred immunosuppressive agents for IMN[5]. In particular, they suggest that CTX should be used as the first line therapy for IMN. It not only reduced the long term events of ESKD or all-cause mortality, but also achieved high proteinuria remission rates that were superior to those achieved with chlorambucil. It was associated with high withdrawal rates, but these again appeared lower than those seen with chlorambucil in direct and indirect comparisons. Chen et al [51] have performed a systematic review and meta-analysis which including RCT found that alkylating agents plus corticosteroids had long-term and short-term benefits for adult IMN, but resulted in more withdrawals or hospitalizations. This result is consistent with our analysis. In recent years, more and more patients have adopted intravenous CTX rather than oral to reduce the side effects of drug.

Calcineurin inhibitors, including tacrolimus and CsA, are also widely used in IMN. Our results demonstrated no clear effect on mortality/ESKD events with these agents, although power was low due to a small number of events. As tacrolimus increases the risk of diabetes via reductions in insulin secretion and inhibition of insulin gene expression stimulus by hyperglycemia [52,53], long term monitoring blood glucose level is important for patients with IMN treated with this agent. A meta-analysis[54] including 21 RCTs was shown that calcineurin inhibitors had better short-term efficacy and safety than CTX, but the CsA has a higher relapse rate. Our analysis indicated that the patients who treated with CsA had hightest relapse rate. So, well-designed clinical trials are needed to further evaluate the long-term efficacy and safety of CNIs.

The data regarding other agents was limited. We found that MMF was consistent with the recommendations from the KDIGO guidelines, which suggests that the monotherapy with MMF should not be used as a first line therapy for of IMN [5]. While the effects of ACTH appear promising, the data on this treatment was provided by only one study with 15 patients published in abstract form and of uncertain reliability. Mizoribine and leflunomide were also assessed in few patients. Further, appropriately powered, high-quality RCTs of these agents would appear worthwhile.

We ranked different treatment strategies for each outcome by SUCRA probabilities and the posterior probabilities. The higher the SUCRA value, the higher the ranking. The results indicated that alkylating agent had best probability in reducing the risk of composite endpoint of mortality or ESKD. In terms of proteinuria remission, ACTH, CTX, tacrolimus and CsA had good probabilities of achieving proteinuria remission, however, only one small trial reported ACTH. Tacrolimus and CsA had best tolerance in all immunosuppressive agents.

Overall, the total remission rate was 59.2% in the patients treated with immunosuppressive therapy, and 32.4% in patients treated with non-immunosuppressive agents. This difference highlights the impact of immunosuppressive therapy in these high risk individuals, but also suggests that better treatments are still required to further increase this remission rate. It is hoped that novel immunsuppressive therapies in development may improve outcomes with less toxicity, and that our recent advances in understanding the role of anti PLA2 receptor antibodies will lead to better targeted treatments. In recent years, new immunosuppressive agents such as rituximab, Tripterygium wilfordii and others have been used in IMN. However, there are no RCTs to assess the efficacy and safety of used for IMN. Tripterygium wilfordii is a traditional Chinese medicine with immunosuppressive effects. It has been reported to be effective in Chinese patients, but the mechanism is not clear [55].

### Advantages and innovations

Previous studies had analyzed the effect of immunosuppressive therapy and (or) non-immunosuppressive therapy only by direct comparison. This study is the first time to comprehensive analysis the effectiveness and tolerance of different treatments for IMN by network meta-analysis, including direct and indirect comparison. The advantage of this new method is to compare a variety of treatment options which used for the same disease and rank the treatments according to the analysis results. Classic meta-analysis often focused on these treatments which common application in clinical, such as: CTX, tacrolimus and cyclosporine. But for the other treatments which RCTs were fewer or minimal application in clinical such as leflunomide, azathioprine, mizoribine, there is no evidence-based research. In this paper, all of the RCTs that met the inclusion criteria were included in the comparisons. For those critical positive results, we also confirmed by direct and indirect comparisons.

### Limitations

Our study used a comprehensive network meta-analysis approach to compare the effectiveness and tolerability of immunosuppressive treatments for idiopathic membranous nephropathy. It has quantitatively assessed and compared different immunosuppressive agents used for IMN[56]. However our study has a number of limitations. First, the quality of the including studies varied, causing significant heterogeneity. Second, the potential for reporting bias exists, which may influence the results. Third, the sample sizes of these studies are relatively small, reducing statistical power.

## Conclusions

Cyclophosphamide and chlorambucil reduce risk of ESKD or death in IMN with nephrotic range proteinuria, but carry substantial toxicity that may be lower for cyclophosphamide. Tacrolimus and cyclosporine increase the possibility of proteinuria remission with less drug withdrawal, but the effects on kidney failure remain uncertain.

## Supporting information

S1 Fig The individual risk of bias data for the included studies. (TIF)

S2 Fig The aggregate risk of bias data for the included studies. (TIF)

S3 Fig The cumulative probability graph for the total mortality or ESKD outcome. The solid line represents the estimated probabilities and the dotted line represents the predicted probabilities. The larger the area under the curve, the higher the ranking.
(TIF)

S4 Fig The cumulative probability graph for the total remission outcome. The solid line represents the estimated probabilities and the dotted line represents the predicted probabilities. The larger the area under the curve, the higher the ranking.
(TIF)

S5 Fig The cumulative probability graph for the withdrawal outcome. The solid line represents the estimated probabilities and the dotted line represents the predicted probabilities. The larger the area under the curve, the higher the ranking.
(TIF)

S6 Fig The result of sensitivity meta-analysis of total mortality or ESKD (excluding low quality studies). (TIF)

S7 Fig The result of sensitivity meta-analysis of total remission (excluding low quality studies). (TIF)

S8 Fig The result of sensitivity meta-analysis of withdrawal (excluding low quality studies). (TIF)

S1 Table Search strategy. (DOCX)

S2 Table Characteristics of studies included in systematic review and meta-analysis. (DOCX)

S1 File PRISMA checklist. (DOC)
