# Supplementary figures and images for: Comparative effectiveness and tolerance of immunosuppressive treatments for idiopathic membranous nephropathy: A network meta-analysis
Source: PLoS One. 2017 Sep 12;12(9):e0184398. doi: 10.1371/journal.pone.0184398 (PMC5595305; doi:10.1371/journal.pone.0184398)

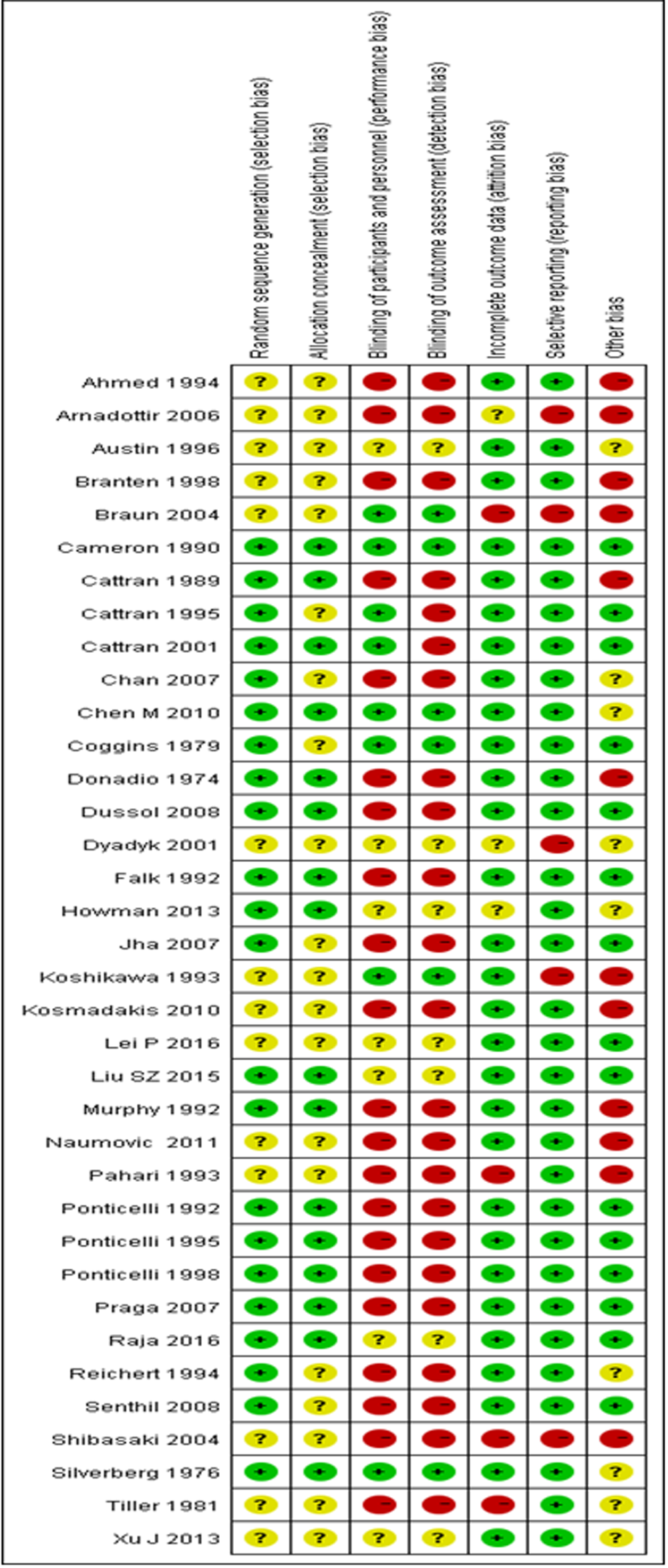

Supplement: S1 Fig — (TIF) [file pone.0184398.s001.tif]

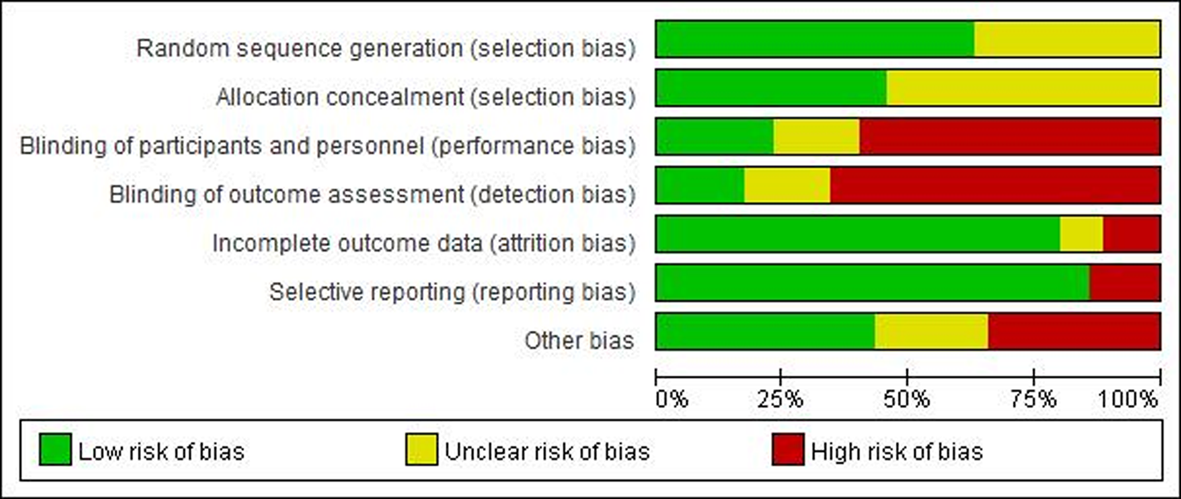

Supplement: S2 Fig — (TIF) [file pone.0184398.s002.tif]

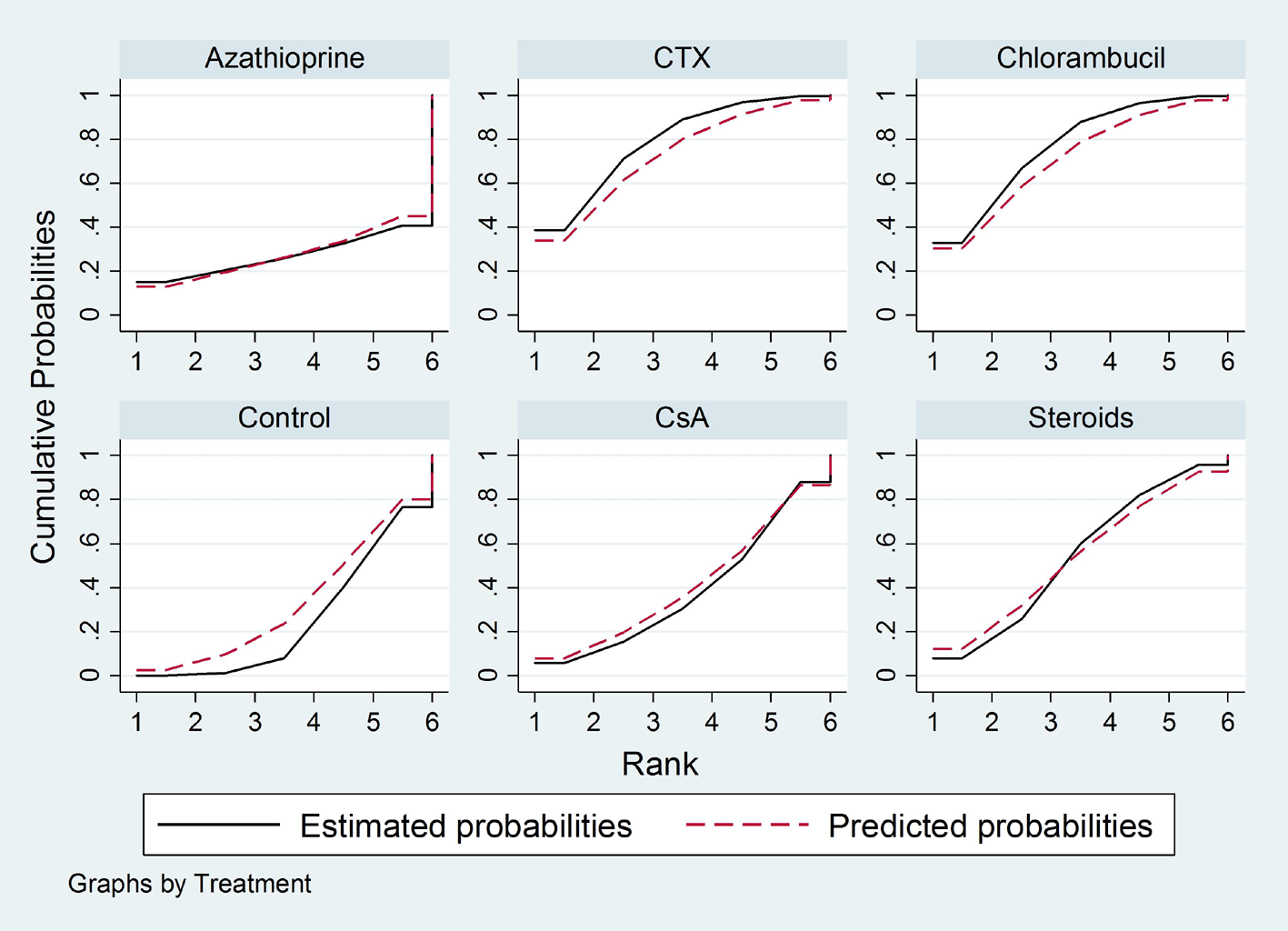

Supplement: S3 Fig — The solid line represents the estimated probabilities and the dotted line represents the predicted probabilities. The larger the area under the curve, the higher the ranking. (TIF) [file pone.0184398.s003.tif]

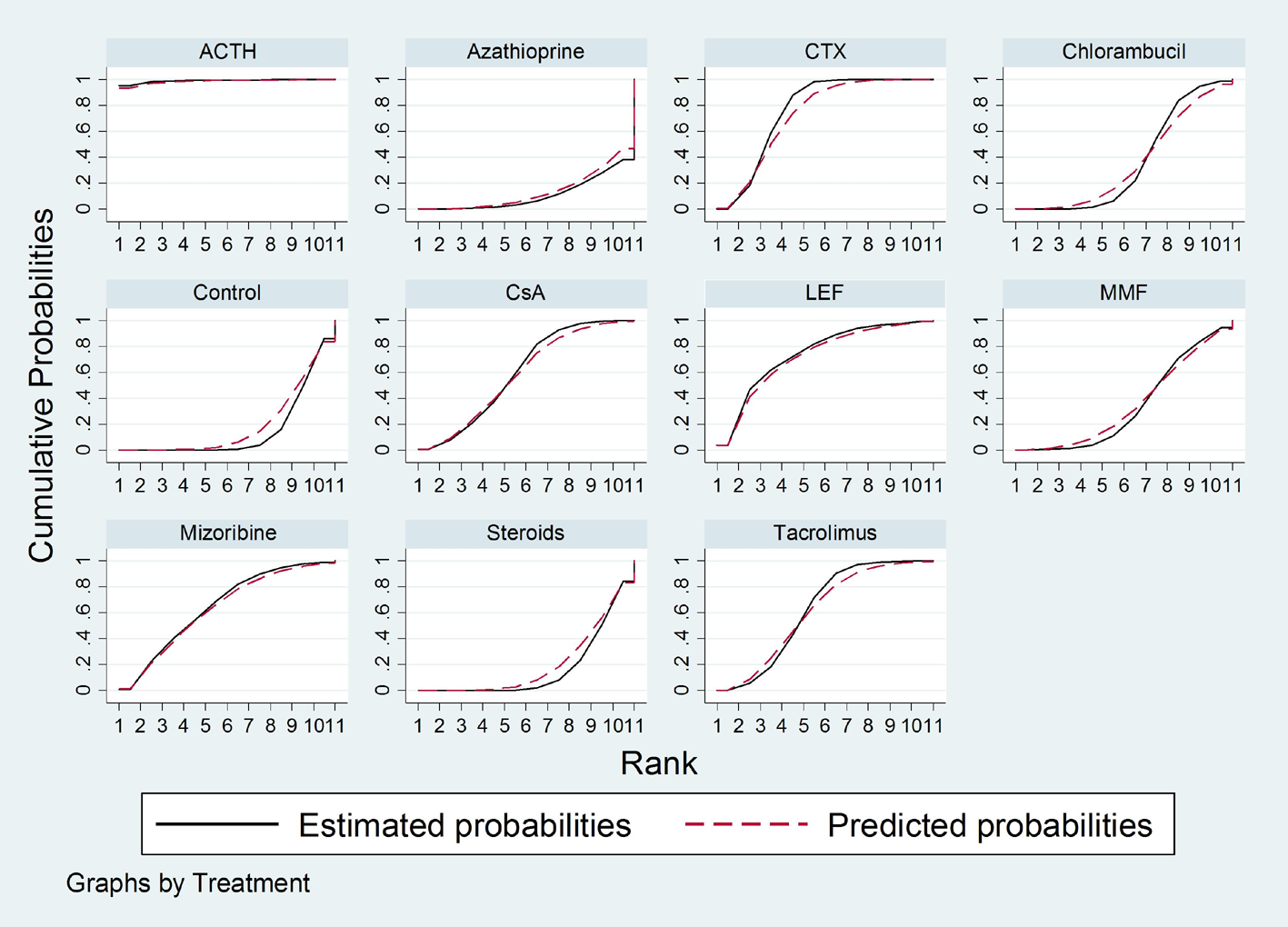

Supplement: S4 Fig — The solid line represents the estimated probabilities and the dotted line represents the predicted probabilities. The larger the area under the curve, the higher the ranking. (TIF) [file pone.0184398.s004.tif]

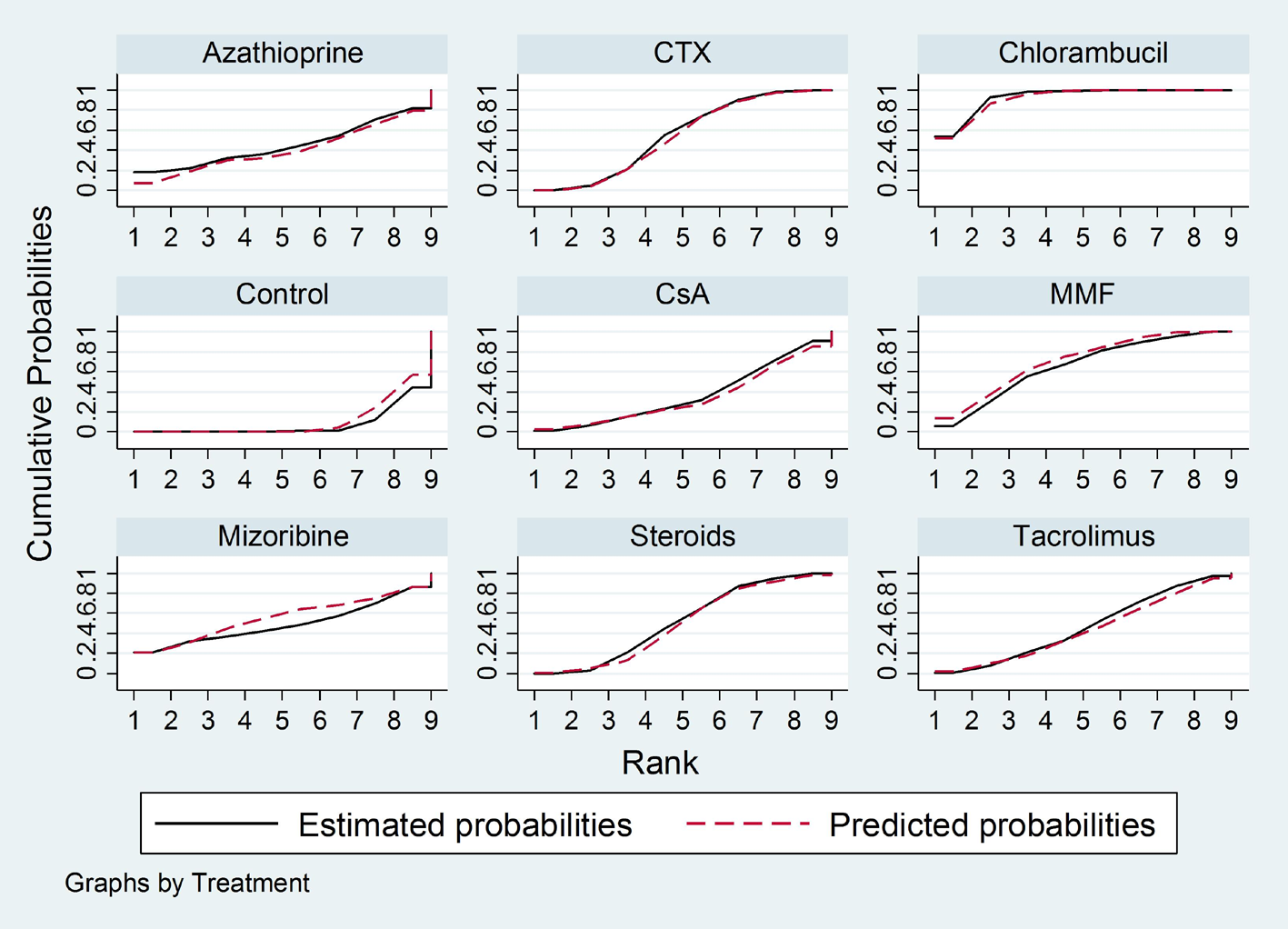

Supplement: S5 Fig — The solid line represents the estimated probabilities and the dotted line represents the predicted probabilities. The larger the area under the curve, the higher the ranking. (TIF) [file pone.0184398.s005.tif]

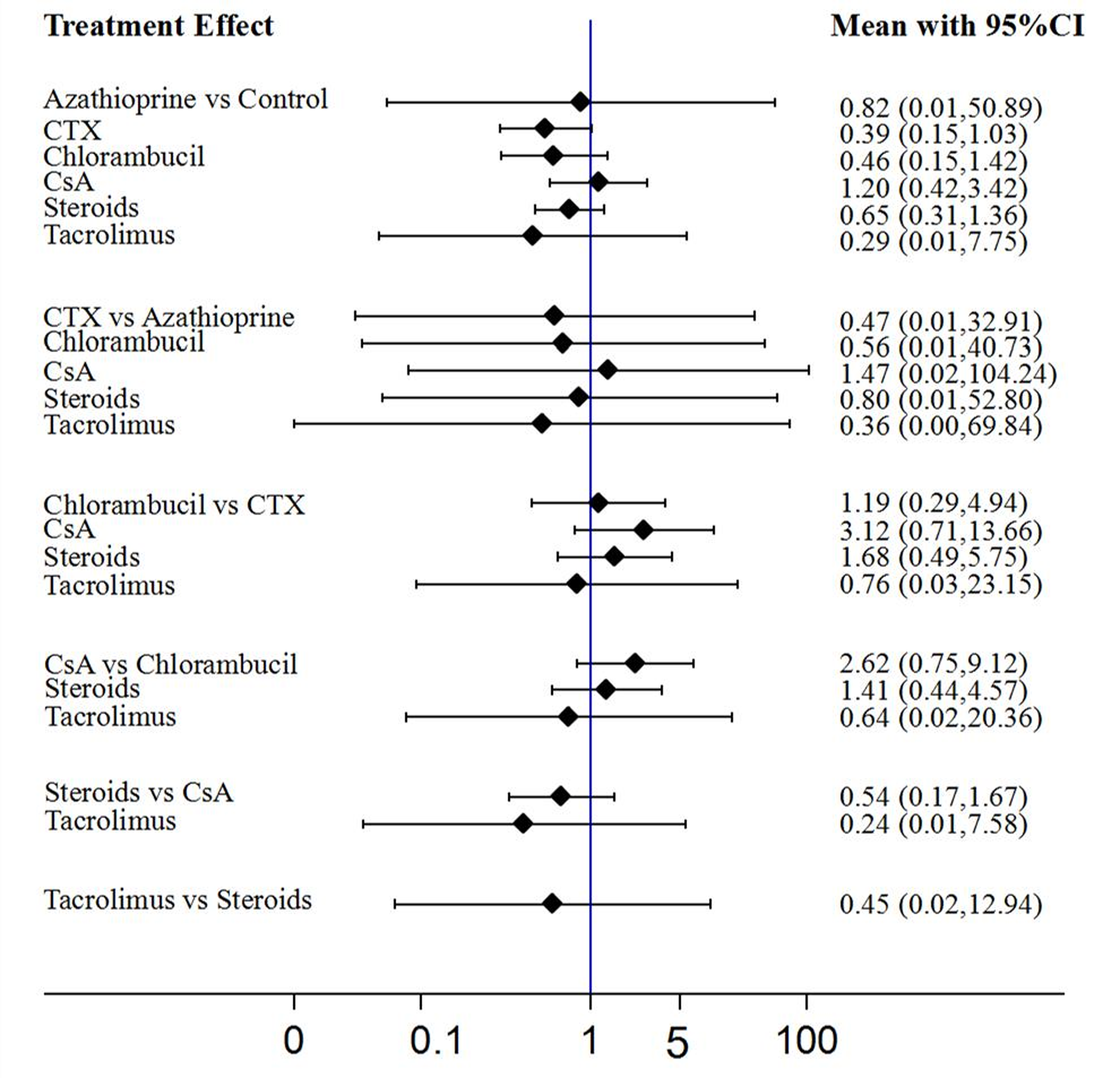

Supplement: S6 Fig — (TIF) [file pone.0184398.s006.tif]

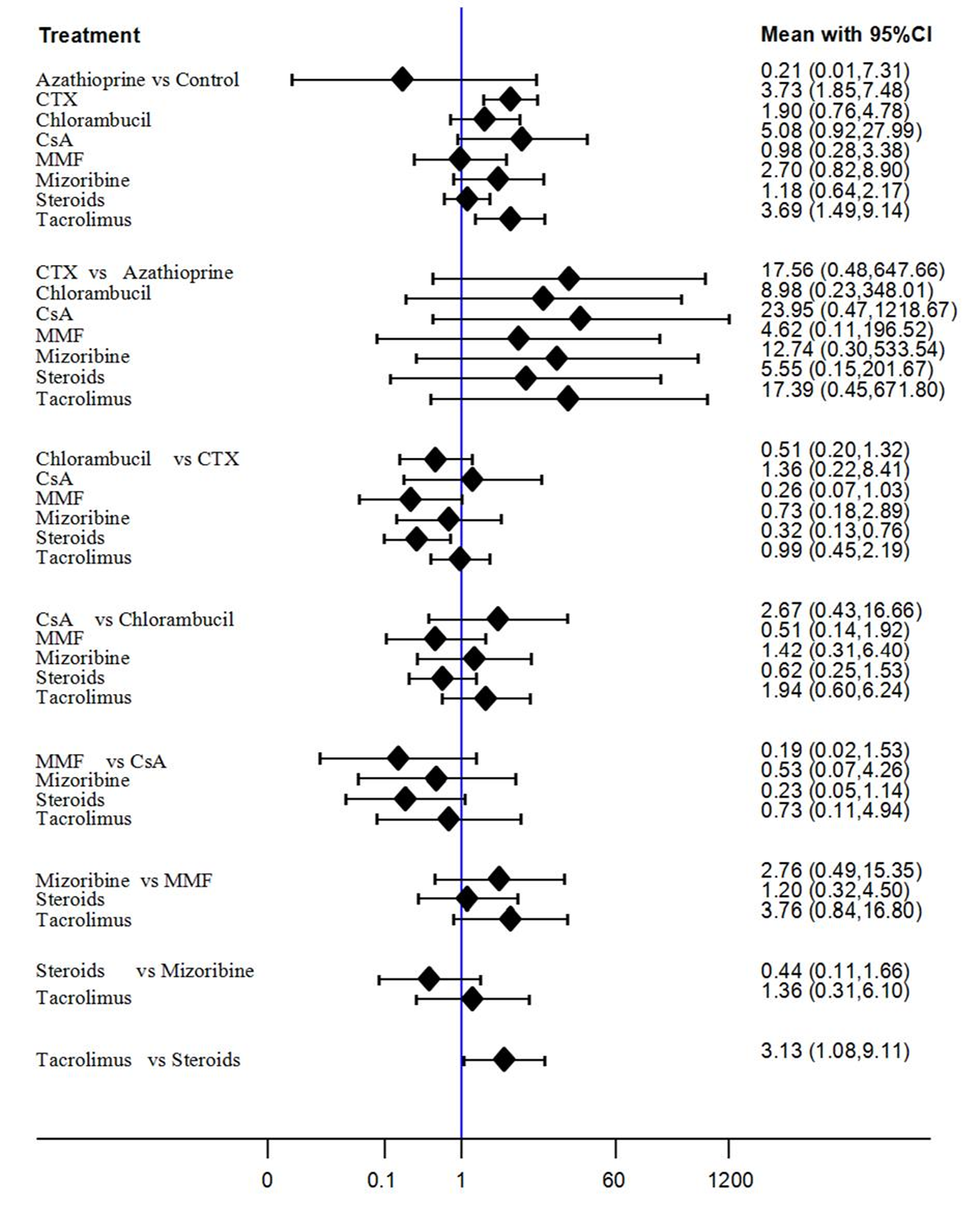

Supplement: S7 Fig — (TIF) [file pone.0184398.s007.tif]

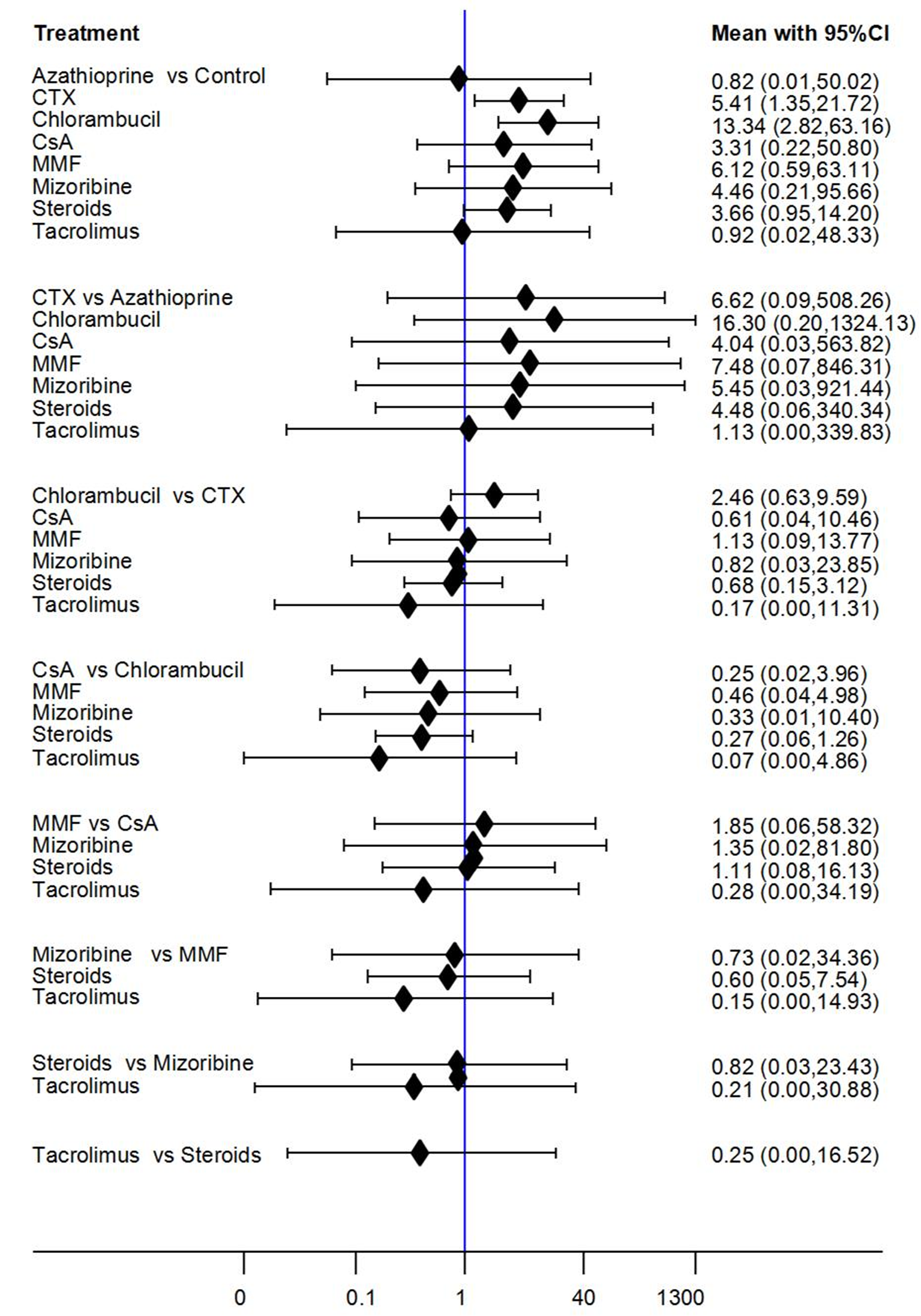

Supplement: S8 Fig — (TIF) [file pone.0184398.s008.tif]
