# Supplementary material for: Comparative effectiveness and tolerance of immunosuppressive treatments for idiopathic membranous nephropathy: A network meta-analysis
Source: PLoS One. 2017 Sep 12;12(9):e0184398. doi: 10.1371/journal.pone.0184398 (PMC5595305; doi:10.1371/journal.pone.0184398)
Supplement: S1 Table — (DOCX) [file pone.0184398.s009.docx]

SEARCH STRATEGY:

**Database Search terms**

CENTRAL via OVID

#1 GLOMERULONEPHRITIS, MEMBRANOUS explode all trees (MeSH)

#2 (glomerulonephritis near membranous)

#3 (membranous near glomerulopathy)

#4 mgn

#5 (nephropathy near membranous)

#6 (extramembranous near glomerulopathy)

#7 (membranous near glomerulonephropathy)

#8 (idiopathic near membranous near glomerulonephritis)

#9 (idiopathic near membranous near nephropathy)

Nephrotic near membranous histology

#10 #1 or #2 or #3 or #4 or #5 or #6 or #7 or #8 or #9

MEDLINE via OVID

1 controlled clinical trial.pt.

2 randomised controlled trial.pt.

3 randomised controlled trials/

4 random allocation/

5 double blind method/

6 single blind method/

7 clinical trial.pt.

8 exp clinical trials/

9 placebos/

10 placebo$.mp.

11 random$.mp.

12 research design/

13 volunteer$.mp.

14 (clin$ adj25 trial$).mp.

15 ((singl$ or doubl$ or trebl$ or tripl$) adj25 (blind$ or mask$)).mp.

16 factorial.mp.

17 cross-over studies/

18 crossover.mp.

19 latin square.mp.

20 (balance$ adj2 block$).mp.

21 (animal not human).sh.

22 glomerulonephritis, membranous/

23 (glomerulonephritis adj2 membranous).mp.

24 membranous glomerulopathy.mp.

25 mgn.mp.

26 (nephropathy adj2 membranous).mp.

27 extramembranous glomerulopathy.mp.

28 membranous glomerulonephropathy.mp.

29 idiopathic membranous glomerulonephritis.mp.

30 idiopathic membranous nephropathy.mp.

31 or/22-30

32 or/1-20

33 32 not 21

34 exp Therapeutics/

35 "TREATMENT".mp.

36 therap$.mp.

37 or/34-36

38 31 and 33 and 37
