# Supplementary material for: Comparative effectiveness and tolerance of immunosuppressive treatments for idiopathic membranous nephropathy: A network meta-analysis
Source: PLoS One. 2017 Sep 12;12(9):e0184398. doi: 10.1371/journal.pone.0184398 (PMC5595305; doi:10.1371/journal.pone.0184398)
Supplement: S2 Table — (DOCX) [file pone.0184398.s010.docx]

**Characteristics of studies included in systematic review and meta-analysis**

| Xu J  (2013) | Methods | Study design: prospective RCT Study duration: 2007-2012 |
| --- | --- | --- |
|  | Participants | Country: China  Setting: single-center  Inclusion criteria: 1) Patients with biopsy-proven IMN with nephrotic syndrome;  2) Age between 18 and 80 years  3) No immunosuppressive treatment in the previous 6 months  Characteristics of the patients at baseline: Proteinuria (g/24 h): treatment group 1 (5.10±2.20); treatment group 2 (5.39 ± 2.51) SCr (mg/dL): treatment group 1 (1.04 ± 0.53); treatment group 2 (0.88 ± 0.26) Number: treatment group 1 (52); treatment group 2 (48) Mean age ± SD (years):  treatment group 1 (57.8±14.8); treatment group 2 (56.3±13.2) Sex (M/F): treatment group 1 (30/22); treatment group 2 (31/17) |
|  | Interventions | Treatment group 1  CTX dosage of 0.5–0.75 g/m2/month lasted 9 months; Prednisone dosages were 1 mg/kg/day Treatment group 2 Tacrolimus dosage of 0.1 mg/kg/day; Prednisone dosage of 0.5 mg/kg/day |
|  | Outcomes | Partial or complete remission  Infection  Abnormal liver function  Incidence of DM |
|  | Methods | Study design: multicenter RCT Study duration: 1998-2008 |
| Howman  (2013) | Participants | Country: UK  Setting: multicenter  Inclusion criteria: 1) Biopsy-proven diagnosis of idiopathic membranous nephropathy  2) Age between 18 and 75 years  3) Serum or plasma creatinine concentration of less than 300 μmol/L together with a 20% or greater decline in excretory renal function  Characteristics of the patients at baseline: Proteinuria (g/24 h): treatment group 1 (10.1±5.3); treatment group 2 (6.8± 4.7); treatment group 3 (9.1±5.3) Ccr (ml/min): treatment group 1 (50 ± 16); treatment group 2 (49 ± 18); treatment group 3 (50 ± 20) Number: treatment group 1 (33); treatment group 2 (36); treatment group 3 (37) Mean age ± SD (years): treatment group 1 (58±12); treatment group 2 (58±11); treatment group 3 (56±16) |
|  | Interventions | Treatment group 1  During months 1, 3, and 5 oral chlorambucil at a starting dose of 0·15 mg/kg per day  Intravenous methyl prednisolone 1 g per day for 3 days then oral prednisolone 0·5 mg/kg per day for 28 days during months 1, 3, and 5. Treatment group 2  Ciclosporin received a starting dose of 5 mg/kg per day  Treatment group 3  Supportive therapy only |
|  | Outcomes | Further 20% decline in excretory renal function from baseline readings  Composite endpoint of mortality or ESRD  Infection |
|  | Methods | Study design: parallel RCT Study duration: 1995-2012 |
| Naumovic  (2011) | Participants | Country: Serbia  Setting: single-center  Inclusion criteria: Patients with biopsy-proven IMN and nephrotic syndrome or renal insufficiency  Characteristics of the patients at baseline: Proteinuria (g/24 h): treatment group 1 (11.6±4.7); treatment group 2 (7.0 ± 2.7) SCr (umol/L): treatment group 1 (124.5 ± 75.9); treatment group 2 (120.5 ± 46.5)  GFR (mL/min): treatment group 1 (80.7±27.5); treatment group 2 (76.2 ±31.3) Number: treatment group 1 (10); treatment group 2 (13) Mean age ± SD (years):  treatment group 1 (39.2±13.1); treatment group 2 (47.5±8.2) Sex (M/F): treatment group 1 (9/1); treatment group 2 (8/3) |
|  | Interventions | Treatment group 1:  CSA: 3 mg/kg/d. During the follow-up, the CSA dose was adjusted to achieve 12-h trough levels of 80-100 ng/mL.  Prednisone: 0.5 mg/kg/d 8 weeks. The dose was gradually reduced to 5 to 10mg/d, and remained unchanged until the end of the treatment. Treatment group 2:  AZA: 1.5-2 mg/kg for 6 months, and afterwards 50 mg/d. AZA was temporarily withdrawn or the dose was reduced if the white cell count fell below 4x109/L  Prednisone: 0.5 mg/kg/d 8 weeks. The dose was gradually reduced to 5 to 10mg/d, and remained unchanged until the end of the treatment tacrolimus dosage of 0.1 mg/kg/day Prednisone dosage of 0.5 mg/kg/day |
|  | Outcomes | Partial or complete remission  Composite endpoint of mortality or ESRD  Withdrawal of treatments  Infection  Bone marrow suppression  Abnormal liver function  Incidence of hypertension |
|  | Methods | Study design: open, parallel RCT  Study duration: before 2010 |
| Kosmadakis (2010) | Participants | Country: Greece  Setting: single-center  Inclusion criteria: biopsy-proven IMN with NS for a period over 6 months and no apparent secondary cause of membranous nephropathy.  Characteristics of the patients at baseline: Proteinuria (g/24 h): treatment group 1 (6.6±1.0); treatment group 2 (7.0 ± 0.7); treatment group 3 (5.2 ± 0.8)  GFR (mL/min): treatment group 1 (81.6±8); treatment group 2 (51.5 ±7); treatment group 3 (65.7 ±5.6)  Albumin (g/dL): treatment group 1 (2.7±0.7); treatment group 2 (2.8 ±0.2); treatment group 3 (2.2 ±0.14) Number: treatment group 1 (10); treatment group 2 (8); treatment group 3 (10) Mean age ± SD (years):  treatment group 1 (50.5±4.9); treatment group 2 (55.4±2.8); treatment group 3 (51.8±5.4) Sex (M/F): treatment group 1 (8/2); treatment group 2 (4/4); treatment group 3 (5/5) |
|  | Interventions | Treatment group 1:  CSA + steroids for 9 months;  Oral CSA: 3-3.5 mg/kg/d; Oral methylprednisolone: 12.5 mg/d  Treatment group 2:  CTX + steroids for 9 months Oral CTX: 2 mg/kg/24 h; Oral methylprednisolone: 1.5 mg/kg/48 h  Treatment group 3:  ACEI for 9 months; Lisinopril |
|  | Outcomes | Partial or complete remission  Infection  Bone marrow suppression |
|  | Methods | Study design: open, parallel RCT  Study duration: 2004-2008 |
| Chen M  (2010) | Participants | Country: China  Setting: multicenter  Inclusion criteria: (1) IMN (stageI–III) proven by renal biopsy; with nephrotic syndrome, which was defined as proteinuria 3.5 g/24 hr, serum albumin 30g/L, edema, and/or hyperlipidemia;  (2) age between 18 and 70 years;   1. initial serum creatinine (Scr) was lower than 221umol/L; 2. willing to give written informed consent and comply with the study protocol; 3. no immunosuppressive treatment within previous 3 months.   Characteristics of the patients at baseline: Proteinuria (g/24 h): treatment group 1 (7.71±3.93); treatment group 2 (7.28± 3.91) SCr (umol/L): treatment group 1 (75.7 ± 22.4); treatment group 2 (85.0 ± 37.5)  GFR (mL/min): treatment group 1 (105.5±28.7); treatment group 2 (97 ±34.3)  Alb (g/L): treatment group 1 (23.1±4.25); treatment group 2 (23.1 ±4.81) Number: treatment group 1 (39); treatment group 2 (34) Mean age ± SD (years):  treatment group 1 (47.2±11.9); treatment group 2 (48.6±11.6) Sex (M/F): treatment group 1 (23/16); treatment group 2 (18/16) |
|  | Interventions | Treatment group 1:  tacrolimus at a dose of 0.1 mg/kg/d  oral prednisone 1mg/kg/d for 4 weeks, tapered gradually, and discontinued by 8months.  Treatment group 2:  oral CTX 100 mg/d for 4 months then reduced by 50 mg/d  oral prednisone 1mg/kg/d for 4 weeks, tapered gradually, and discontinued by 8months. |
|  | Outcomes | Partial or complete remission  Withdrawal of treatments  Infection  Abnormal liver function  Incidence of hypertension  Incidence of DM |
|  | Methods | Study design: open, parallel RCT Study duration: before 2008 |
| Senthil  (2008) | Participants | Country: India  Setting: single-center  Inclusion criteria: Adult patients with nephrotic syndrome and biopsy-proven IMN. Kidney biopsies were evaluated by light microscopy and immunofluorescence  Characteristics of the patients at baseline: Urine protein:creatinine ratio (mg/mg): treatment group 1 (4.68±1.82); treatment group 2 (4.95 ± 1.65) Alb(g/dl): treatment group 1 (2.7 ± 0.6); treatment group 2 (2.6 ± 0.5)  GFR (mL/min): treatment group 1 (86±12.6); treatment group 2 (82 ±11.8) Number: treatment group 1 (11); treatment group 2 (10) Mean age ± SD (years): NS Sex (M/F): NS |
|  | Interventions | Treatment group 1:  MMF + steroids MMF: 2 g/day in 2 divided doses for 6 months; Prednisolone: 0.5 mg/kg/d for 8-12 wk.  Treatment group 2:  CTX + steroids (3 cycles for 6 months) Methylprednisolone: IV 1 g/d for 3 consecutive days followed by oral prednisolone 0.5 mg/kg/d for 27 days. The cumulative prednisolone dose was 2 ± 0.4 g; Oral CTX: 2 mg/kg/d for 30 days |
|  | Outcomes | Partial or complete remission  Withdrawal of treatments  Infection |
|  | Methods | Study design: open, parallel RCT Study duration: 2004-2008 |
| Dussol  (2008) | Participants | Country: France  Setting: multicentre  Inclusion criteria: Idiopathic biopsy-proven MGN, age older than 18 years, nephrotic syndrome (proteinuria >3g/day with hypoalbuminemia with albumin level <3 g/dL and serum creatinine level< 2.26 mg/dL  Characteristics of the patients at baseline: Proteinuria (g/24 h): treatment group 1 (6.2±3.5); treatment group 2 (9.5 ± 5.8) SCr (mg/dL): treatment group 1 (1.01 ± 0.34); treatment group 2 (1.09 ± 0.39)  GFR (mL/min): treatment group 1 (92.1±29.8); treatment group 2 (80.7 ±25.4)  Alb (g/L): treatment group 1 (23.2±7.3); treatment group 2 (20.2 ±6.0) Number: treatment group 1 (19); treatment group 2 (17) Mean age ± SD (years):  treatment group 1 (47.8±15.2); treatment group 2 (55.9±15.2) Sex (M/F): treatment group 1 (17/2); treatment group 2 (15/2) |
|  | Interventions | Treatment group 1:  MMF: 250 mg/d, progressively increased by 250 mg every other day to 2 g/d for 12 months. MMF therapy was then progressively stopped in 15 days. Mean dose of MMF was 1,850 mg.  Treatment group 2:  Renin-angiotensin blockers, statins, low-salt and low-protein diet, and diuretics in case of oedema |
|  | Outcomes | Partial or complete remission  Withdrawal of treatments  Infection  Incidence of hypertension |
|  | Methods | Study design: open, parallel RCT Study duration: 2003-2006 |
| Praga  (2007) | Participants | Country: Spain  Setting: multicentre  Inclusion criteria: Patients with biopsy-proven MGN and preserved renal function, and with persistent nephrotic syndrome for more than 9 months despite treatment with angiotensin-converting enzyme inhibitors (ACEI) or angiotensin receptor blockers (ARB).  Characteristics of the patients at baseline: Proteinuria (g/24 h): treatment group 1 (7.2±3.3); treatment group 2 (8.4 ± 5.4) SCr (mg/dL): treatment group 1 (0.98 ± 0.2); treatment group 2 (1.1 ± 0.3)  GFR (mL/min): treatment group 1 (104±26); treatment group 2 (107 ±63) Number: treatment group 1 (25); treatment group 2 (23) Mean age ± SD (years):  treatment group 1 (43.7±12.1); treatment group 2 (50.1±12.2) Sex (M/F): treatment group 1 (20/5); treatment group 2 (20/3) |
|  | Interventions | Treatment group 1:  Tacrolimus: 0.05 mg/kg/d, divided into two daily doses at 12-h interval. Later doses were adjusted to achieve a whole blood 12-h trough level between 3 and 5 ng/mL.  Treatment group 2:  No specific immunosuppressive treatment |
|  | Outcomes | Partial or complete remission  Composite endpoint of mortality or ESRD  Infection  Incidence of hypertension  Incidence of DM |
|  | Methods | Study design: open, parallel RCT Study duration: 1993-1995 |
| Jha  (2007) | Participants | Country: India  Setting: single-center  Inclusion criteria: adult (age >16 yr) patients with nephrotic syndrome caused by biopsy-proven IMN of at least 6 mo duration.  Characteristics of the patients at baseline: Proteinuria (g/24 h): treatment group 1 (6.11±2.5); treatment group 2 (5.91 ± 2.2) SCr (mg/dL): treatment group 1 (1.21 ± 0.31); treatment group 2 (1.17 ± 0.22)  GFR (mL/min): treatment group 1 (89±26); treatment group 2 (84±22) Number: treatment group 1 (47); treatment group 2 (46) Mean age ± SD (years):  treatment group 1 (38.0±13.6); treatment group 2 (37.2±12.4) |
|  | Interventions | Treatment group 1:  IV methylprednisolone 1 g/d for 3 consecutive days followed by oral prednisolone 0.5 mg/kg/d for 27 d in the first, third, and fifth months; Oral CTX 2 mg/kg/d in the second, fourth, and sixth months.  Treatment group 2:  Supportive therapy that consisted of dietary sodium restriction, diuretics, and antihypertensive agents |
|  | Outcomes | Partial or complete remission  Composite endpoint of mortality or ESRD  Withdrawal of treatments  Infection |
|  | Methods | Study design: open, parallel RCT Study duration: before 2007 |
| Chan  (2007) | Participants | Country: China  Setting: single-center  Inclusion criteria: Aged 18–65 years; diagnosed idiopathic MN confirmed by renal biopsy within the past 6 months; proteinuria of >3 g/day that did not show spontaneous improvement; serum creatinine below 300 mmol/L  Characteristics of the patients at baseline: Proteinuria (g/24 h): 5.7 ± 2.7  Serum albumin (g/L): 26.5 ± 7.5  SCr (µmol/L): treatment group 1 (103.3 ± 48.7); treatment group 2 (85.7 ±31.8)  GFR (mL/min): treatment group 1 (87.1 ± 38.5); treatment group 2 (101.8±40.6) Number: treatment group 1 (11); treatment group 2 (9) Mean age ± SD (years): 49.5±13.5 Sex (M/F): 13/7 |
|  | Interventions | Treatment group 1:  MMF: 1 g bid was given for 6 months.  Oral prednisolone: started at 0.8 mg/kg/d, then tapered by 5 mg/d Treatment group 2:  Methylprednisolone IV1 g daily for 3 days, followed by prednisolone 0.4 mg/kg per day for 3 weeks, then 0.2 mg/kg per day till the end of the month, alternating with chlorambucil 0.2 mg/kg per day for 1 month, for a total  duration of 6 months. |
|  | Outcomes | Partial or complete remission  Withdrawal of treatments  Infection  Bone marrow suppression  Incidence of hypertension  Incidence of DM |
|  | Methods | Study design: open, parallel RCT Study duration: 1996-2001 |
| Shibasaki  (2004) | Participants | Country: Japan  Setting: multicentre  Inclusion criteria: Patients with primary NS not showing a satisfactory response to corticosteroid therapy for 3 months (steroid resistant), but who had stable laboratory and clinical findings while receiving a daily maintenance dose of 20mg prednisolone-equivalent a day (including zero dosage) and who gave consent for participation in this study.  Characteristics of the patients at baseline: Proteinuria: NS  Serum albumin: NS  SCr (mg/dL): < 2.0  GFR (mL/min): ≥ 40 Number: treatment group 1 (14); treatment group 2 (11) |
|  | Interventions | Treatment group 1:  Mizoribine: 50 mg, 3 times/d. after meals  No particular restriction was placed on the use of corticosteroids during the study period Treatment group 2:  Conservative therapy |
|  | Outcomes | Partial or complete remission |
|  | Methods | Study design: parallel RCT  Study duration: before 2001 |
| Cattran  (2001) | Participants | Country: Canada, USA  Setting: multicentre  Inclusion criteria: Patients with biopsy-proven steroid-resistant IMN and nephrotic-range proteinuria. All patients must have failed to achieve remission of their proteinuria after a minimum of 8 weeks of prednisone treatment at ≥1 mg/kg/d  Characteristics of the patients at baseline: Proteinuria (g/24 h): treatment group 1 (9.7 ± 5.3); treatment group 2 (8.8±4.7)  Serum albumin (g/L): treatment group 1 (28 ± 6); treatment group 2 (27 ± 6)  SCr (mg/dL): treatment group 1 (1.3 ± 0.5); treatment group 2 (1.1 ± 0.3)  GFR (mL/min/1.73 m²): treatment group 1 (95 ± 37); treatment group 2 (90 ± 27) Number: treatment group 1 (28); treatment group 2 (23) Mean age ± SD (years):  treatment group 1 (47 ± 11); treatment group 2 (49 ± 14)  Sex (M/F): treatment group 1 (26/2); treatment group 2 (16/7) |
|  | Interventions | Treatment group 1:  CSA: started at a dose of 3.5 mg/kg/d in 2 equal doses at 12-hour intervals. Adjustments in dosages were made to achieve a whole-blood 12-hour trough level measured by monoclonal assay between 125 and 225 mg/L.  Prednisone: 0.15 mg/kg/d up to a maximum dose of 15 mg. This was reduced after 26 weeks by thirds at 4-week intervals and was stopped after 8 weeks. Treatment group 2:  Placebo: started at a dose of 0.035 mL/kg/d;  Prednisone: 0.15 mg/kg/d up to a maximum dose of 15 mg. This was reduced after 26 weeks by thirds at 4-week intervals and was stopped after 8 weeks. |
|  | Outcomes | Partial or complete remission  Composite endpoint of mortality or ESRD  Incidence of hypertension |
|  | Methods | Study design: parallel RCT Study duration: before 1998 |
| Ponticelli  (1998) | Participants | Country: Italy  Setting: multicentre  Inclusion criteria: Patients with biopsy-proven IMN with nephrotic syndrome  Characteristics of the patients at baseline: Proteinuria (g/24 h): treatment group 1 (7.96 ± 5.19); treatment group 2 (6.85 ± 3.51)  SCr (mg/dL): treatment group 1 (1.06 ± 0.27); treatment group 2 (1.04 ± 0.27) Number: treatment group 1 (50); control group 2 (45)  Mean age, range (years): treatment group 1 (50, 18-65); control group 2 (48, 17-55)  Sex (M/F): treatment group 1 (37/13); control group 2 (29/16) |
|  | Interventions | Treatment group 1:  Methylprednisolone: 1 g IV on 3 consecutive days and then 0.4 mg/kg/d given orally for 27 d, in a single morning dose  Chlorambucil: 0.2 mg/kg/d, orally for 1 month. The total duration of treatment, therefore, was 6 mo for both groups; 3 mo with the same doses of methylprednisolone and 3 mo with either of the two cytotoxic drugs. Treatment group 2:  Methylprednisolone: 1 g IV on 3 consecutive days and then 0.4 mg/kg/d given orally for 27 d, in a single morning dose  Oral CTX: 2.5 mg/kg/d. |
|  | Outcomes | Partial or complete remission  Composite endpoint of mortality or ESRD  Withdrawal of treatments  Infection  Incidence of DM |
|  | Methods | Study design: parallel RCT Study duration: 1989-1996 |
| Branten  (1998) | Participants | Country: Netherlands  Setting: NS  Inclusion criteria: Patients with biopsy-proven IMN with nephrotic syndrome and deteriorating kidney function  Characteristics of the patients at baseline: Proteinuria (g/24 h): treatment group 1 (9 ± 2.6); treatment group 2 (11 ±5.3)  SCr (umol/L): treatment group 1 (219 ± 73); treatment group 2 (274 ± 126)  GFR (mL/min): treatment group 1 (46 ± 17); treatment group 2 (43 ± 23)  Alb (g/L): treatment group 1 (22 ± 5.6); treatment group 2 (22 ±6.0)  Number: treatment group 1 (15); treatment group 2 (17)  Mean age ± SD (years):  treatment group 1 (51 ± 12); treatment group 2 (53 ± 14)  Sex (M/F): treatment group 1 (15/0); treatment group 2 (15/2) |
|  | Interventions | Treatment group 1:  Steroids: 1g IV methylprednisolone on 3 consecutive days, followed by oral prednisone 0.5 mg/kg/d, months 1, 3 and 5  Chlorambucil: 0.15 mg/kg/d months 2, 4 and 6  Treatment group 2:  Oral CTX: 1.5-2.0 mg/kg/d for 1 year  Steroids in a comparable dose |
|  | Outcomes | Partial or complete remission  Composite endpoint of mortality or ESRD  Withdrawal of treatments  Infection  Bone marrow suppression |
|  | Methods | Study design: RCT Study duration: NS |
| Austin  (1996) | Participants | Country: USA  Setting: NS  Inclusion criteria: Patients with IMN  Characteristics of the patients at baseline: Proteinuria: NS  Serum albumin: NS  SCr (µmol/L): NS  GFR: 24 to 156 mL/min  Number: treatment group 1 (17); treatment group 2 (14) |
|  | Interventions | Treatment group 1:  IV CTX: (0.5.0 g/m² every other month)  Prednisone: 40 mg/m² every other day for 2 months tapered to 10 mg/m² Treatment group 2:  Prednisone: 40 mg/m² every other day for 2 months tapered to 10 mg/m² |
|  | Outcomes | Partial or complete remission  GFR |
|  | Methods | Study design: open, parallel RCT Study duration: 1976-1983 |
| Ponticelli  (1995) | Participants | Country: Italy  Setting: multicentre  Inclusion criteria: Patients with biopsy-proven IMN with nephrotic syndrome  Characteristics of the patients at baseline: Proteinuria (g/24 h): treatment group 1 (6.18±2.98); treatment group 2 (5.30 ± 2.84) SCr (umol/L): treatment group 1 (93.8 ± 21.5); treatment group 2 (93.1 ± 25.3) Number: treatment group 1 (42); treatment group 2 (39) Range (years):  treatment group 1 43.5(15-70); treatment group 2 42(16-74) Sex (M/F): treatment group 1 (34/8); treatment group 2 (29/10) |
|  | Interventions | Treatment group 1:  Methylprednisolone: 3 cycles of IV 1 g on 3 consecutive days and then 0.4 mg/kg/d given orally for 27 days, in a single morning dose  Oral chlorambucil: 0.2 mg/kg/d Treatment group 2:  No specific immunosuppressive treatment |
|  | Outcomes | Partial or complete remission  Composite endpoint of mortality or ESRD  Withdrawal of treatments  Infection  Incidence of DM |
|  | Methods | Study design: parallel RCT Study duration: before 1994 |
| Cattran  (1995) | Participants | Country: Canada  Setting: multicenter  Inclusion criteria: All recent (≤24 months) biopsy-proven cases of membranous nephropathy in patients aged 18 to 65 with proteinuria ≥1 g/day were eligible for the 12 month observation section of the study.  Characteristics of the patients at baseline: Proteinuria (g/24 h): treatment group 1 (11.5, 9-18); treatment group 2 (12.8, 4-21) SCr (umol/L): treatment group 1 (186 ± 65); treatment group 2 (204 ± 81)  GFR (mL/min): treatment group 1 (51±20); treatment group 2 (46±16) Number: treatment group 1 (9); treatment group 2 (8) Median age, range (years): treatment group 1 (44, 22-59); treatment group 2 (40, 20-61  Sex (M/F): treatment group 1 (8/1); treatment group 2 (6/2) |
|  | Interventions | Treatment group 1:  CSA: 100 mg/mL, was initiated at 3.5 mg/kg/d taken in 2 divided doses, and periodic adjustments were made as necessary to achieve a 12-hour trough level of between 110 and 170 ng/mL.  Treatment group 2:  Placebo: made of the identical carrier except CSA was excluded. It was initially prescribed at 0.035 mL/kg/d, taken in 2 divided quantities with periodic arbitrary adjustments in dose to match the CSA group |
|  | Outcomes | Composite endpoint of mortality or ESRD  Withdrawal of treatments |
|  | Methods | Study design: open, parallel RCT  Study duration: 1989-1992 |
| Reichert  (1994) | Participants | Country: Netherlands  Setting: single-center  Inclusion criteria: patients were required to have a nephrotic syndrome with biopsy-proven IMN and deteriorating renal function Characteristics of the patients at baseline: Proteinuria (g/24 h): treatment group 1 (8.5±2.5); treatment group 2 (9.8 ± 4.8) SCr (umol/L): treatment group 1 (260 ± 112); treatment group 2 (218 ± 85)  Alb (g/L): treatment group 1 (22.9±6.4); treatment group 2 (25.9 ±9.7) Number: treatment group 1 (9); treatment group 2 (9) Mean age, range (years): treatment group 1 (45, 31-65); treatment group 2 (49,24-65)  Sex (M/F): treatment group 1 (9/0); treatment group 2 (8/1) |
|  | Interventions | Treatment group 1:  Oral chlorambucil: 0.15 mg/kg/d in months 2, 4, and 6  Prednisone: 3 IV pulses of 1 g of methylprednisolone followed by oral prednisone at 0.5 mg/kg/d in months 1, 3, and 5) Treatment group 2:  IV CTX: 750 mg/m² body surface area once every month for 6 months  Methylprednisolone: (3 IV 1 g pulses in months 1, 3, and 5) |
|  | Outcomes | Partial or complete remission  Composite endpoint of mortality or ESRD  Withdrawal of treatments  Infection  Bone marrow suppression |
|  | Methods | Study design: parallel RCT  Study duration: before 1989 |
| Ponticelli  (1992) | Participants | Country: Italy  Setting: multicentre  Inclusion criteria: Patients with biopsy-proven IMN with nephrotic syndrome  Characteristics of the patients at baseline: Proteinuria (g/24 h): treatment group 1 (7.6 ± 4.2); treatment group 2 (7.0±4.1)  SCr (mg/dL): treatment group 1 (1.0 ± 0.3); treatment group 2 (1.0 ± 0.3)  Number: treatment group 1 (45); treatment group 2 (47)  Mean age, range (years): treatment group 1 (46, 14-65); treatment group 2 (47,14-64)  Sex (M/F): treatment group 1 (32/13); treatment group 2 (27/20) |
|  | Interventions | Treatment group 1:  Methylprednisolone: 3 cycles of IV 1 g on 3 consecutive days and then 0.4mg/kg/d given orally for 27 days, in a single morning dose  Oral chlorambucil: 0.2 mg/kg/d  Treatment group 2:  IV methylprednisolone: 1 g on 3 consecutive days at the beginning of treatment and again 2 and 4 months  Oral methylprednisolone: 0.4 mg/kg every other day, except during the period of IV administration, for six months |
|  | Outcomes | Partial or complete remission  Composite endpoint of mortality or ESRD  Withdrawal of treatments  Infection  Bone marrow suppression  Abnormal liver function |
|  | Methods | Study design: open, parallel RCT  Study duration: 1986-1990 |
| Falk  (1992) | Participants | Country: USA  Setting: multicenter  Inclusion criteria: Patients with biopsy-proven progressive IMN with either deteriorating kidney function or persistent proteinuria associated with morbid complications  Characteristics of the patients at baseline: Proteinuria (g/24 h): treatment group 1 (12.4 ± 9.9); treatment group 2 (11.1 ± 6.7)  SCr (mg/dL): SCr (mg/dL): treatment group 1 (2.3 ± 1.0); treatment group 2 (2.7 ± 1.6) Number: treatment group 1 (13); treatment group 2 (13)  Mean age ± SD (years): treatment group 1 (43.3 ± 14.8); treatment group 2 (46.0±13.7)  Sex (M/F): treatment group 1 (9/4); treatment group 2 (7/6) |
|  | Interventions | Treatment group 1:  IV CTX in conjunction with a 3-day course of pulse methylprednisolone and alternate-day corticosteroids  Treatment group 2:  oral 2.0 mg/kg prednisone on alternate days for 8 weeks, and then tapered by 25%/dose/wk over 4 weeks |
|  | Outcomes | Composite endpoint of mortality or ESRD  Withdrawal of treatments |
|  | Methods | Study design: parallel RCT  Study duration: 1981-1985 |
| Cameron  (1990) | Participants | Country: UK  Setting: multicentre  Inclusion criteria: Patients with biopsy-proven IMN with nephrotic syndrome  Characteristics of the patients at baseline: Proteinuria (g/24 h): treatment group (10.8 ± 5.9); control group (10.4 ± 5.3)  SCr (umol/L): treatment group (114 ± 42); control group (115 ± 43)  GFR (mL/min): treatment group (87 ± 30); control group (89 ± 34)  Alb (g/L): treatment group (26 ± 6); group (25 ± 5)  Number: treatment group (52); control group (51)  Mean age ± SD (years):  treatment group (45 ± 11.6); control group (44 ± 12.1)  Sex (M/F): treatment group (43/9); control group (43/8) |
|  | Interventions | Treatment group 1:  Prednisolone: 125 mg was given every alternate day for 8 weeks. Patients who weighted more than 80 kg received 150 mg on alternative days  Treatment group 2:  Placebo: identical tablets as prednisolone for 8 weeks |
|  | Outcomes | Partial or complete remission  Composite endpoint of mortality or ESRD  Withdrawal of treatments  Infection |
|  | Methods | Study design: open, parallel RCT  Study duration: 1977-1985 |
| Cattran  (1989) | Participants | Country: Canada  Setting: single-center  Inclusion criteria: Patients with biopsy-proven IMN and nephrotic syndrome  Characteristics of the patients at baseline: Proteinuria (g/24 h): treatment group 1 (6.9±0.8); treatment group 2 (5.2 ± 0.8) SCr (umol/L): treatment group 1 (120 ± 10); treatment group 2 (103 ± 9)  GFR (mL/sec): treatment group 1 (1.3±0.08); treatment group 2 (1.5 ±0.08)  Alb (g/L): treatment group 1 (27 ± 1.3); treatment group 2 (30 ± 1) Number: treatment group 1 (81); treatment group 2 (77)  Median age, range (years): treatment group 1 (46, 18-77); treatment group 2 (45, 16-83)  Sex (M/F): treatment group 1 (61/20); treatment group 2 (44/33) |
|  | Interventions | Treatment group 1:  Prednisone: 45 mg/m² in a single dose on alternate days for 6 months. The cumulative dose was 0.6 ± 0.05 mg/kg/d Treatment group 2:  No specific treatment for 6 months |
|  | Outcomes | Partial or complete remission  Composite endpoint of mortality or ESRD  Withdrawal of treatments  Incidence of DM |
|  | Methods | Study design: parallel RCT Study duration: before 1976 |
| Silverberg  (1976) | Participants | Country: Canada  Setting: multicenter  Inclusion criteria: Patients with biopsy-proven IMN with nephrotic syndrome  Characteristics of the patients at baseline: Proteinuria (g/24 h): treatment group 1 (12.2±4.9); treatment group 2 (9.1 ± 5.9) SCr (mg/dL): treatment group 1 (1.1 ± 0.4); treatment group 2 (1.5 ± 0.5)  GFR (mL/min): treatment group 1 (95 ± 37); treatment group 2 (74 ± 22)  Alb (g/L): treatment group 1 (24 ± 5); treatment group 2 (25 ± 3)  Number: treatment group 1(5); treatment group 2 (4)  Mean age ± SD (years): treatment group1 (41 ± 15); treatment group 2 (45 ± 18)  Sex (M/F): treatment group1 (3/2); treatment group 2 (3/1) |
|  | Interventions | Treatment group 1:  AZA: 2.5 mg/kg/d (in 50 mg tablets) once-a-day for 1 year  Treatment group 2:  Placebo: similar number of placebo tablets as AZA |
|  | Outcomes | Partial or complete remission  Composite endpoint of mortality or ESRD |
|  | Methods | Study design: open, parallel RCT  Study duration: 1971-1973 |
| Donadio  (1974) | Participants | Country: USA  Setting: single-center  Inclusion criteria: Patients with biopsy-proven IMN with nephrotic syndrome  Characteristics of the patients at baseline: Proteinuria (g/24 h): treatment group 1(7.8, 2-16.6); treatment group 2 (7.6, 2-12.1)  Alb (g/L): treatment group 1 (27, 19-34); treatment group 2 (23, 16-37).  SCr (mg/dL): treatment group 1(1.2, 0.8-1.9); treatment group 2 (1.1, 0.8-2.2)  GFR (mL/min): treatment group 1 (75, 44-117); treatment group 2 (80.6, 33-112)  Number: treatment group (11); control group (11)  Mean age, range (years): treatment group 1 (males: 41, 25-74; females: 48.5, 40-59); treatment group 2 (males: 47.6, 34-69; females: 41, 26/65)  Sex (M/F): treatment group 1 (9/2); treatment group 2 (8/3) |
|  | Interventions | Treatment group 1:  Oral CTX: 1.5 to 2.5mg/kg/d (mean: 1.8) for 1 year. Treatment group 2:  No treatment |
|  | Outcomes | Partial or complete remission  Withdrawal of treatments |
|  | Methods | Study design: parallel RCT Study duration: before 2006 |
| Arnadottir  (2006) | Participants | Country: Iceland; Sweden  Setting: multicenter  Inclusion criteria: Patients with biopsy-proven IMN with nephrotic syndrome  Characteristics of the patients at baseline: SCr (µmol/L): treatment group (107); control group (104)  Number: treatment group 1 (15); treatment group 2 (15) |
|  | Interventions | Treatment group 1:  ACTH: SC 1.0 mg qw, 0.75 mg biw or 1.0 mg biw for 9 months  Treatment group 2:  No specific treatment |
|  | Outcomes | Partial or complete remission |
|  | Methods | Study design: parallel RCT Study duration: before 2004 |
| Braun  (2004) | Participants | Country: Germany  Inclusion criteria: Patients with biopsy-proven IMN with nephrotic syndrome  Characteristics of the patients at baseline: Proteinuria (g/24 h): NS  SCr (umol/L): NS  GFR (mL/min): NS  Number: treatment group 1 (31); treatment group 2 (44); treatment group 3 (22) |
|  | Interventions | Treatment group 1:  methyl-prednisolone and Chlorambucil alternating monthly Treatment group 2:  prednisolone and cyclosporine A  Treatment group 3:  symptomatic treatment |
|  | Outcomes | Partial or complete remission |
|  | Methods | Study design: RCT Study duration: before 2001 |
| Dyadyk  (2001) | Participants | Country: Ukraine  Setting: NS  Inclusion criteria: Patients with biopsy-proven IMN  Characteristics of the patients at baseline: Proteinuria (g/24 h): NS  Serum albumin (g/L): NS  SCr (mg/dL): NS  GFR (mL/min): NS  Number: treatment group 1 (16); treatment group 2 (16)  Mean age ± SD (years): NS  Sex (M/F): treatment group 19/13 |
|  | Interventions | Treatment group 1:  CTX: Initial dose:1.5 to 3.5 mg/kg/d  Mean treatment duration: 5.8 months Treatment group 2:  Azathioprine: Initial dose: 1.4 to 2.0 mg/kg/d  Mean treatment duration: 6.6 months |
|  | Outcomes | Partial or complete remission |
|  | Methods | Study design: open, parallel RCT  Study duration: 1978-1986 |
| Murphy  (1992) | Participants | Country: Australia  Setting: multicentre  Inclusion criteria: Patients with biopsy-proven IMN and nephrotic syndrome  Characteristics of the patients at baseline: Proteinuria (g/24 h): treatment group 1(5.0, 0.9-13); treatment group 2 (3.9, 0.5-12)  Serum albumin (g/L): treatment group 1(28, 16-42); treatment group 2 (30, 19-41)  SCr (µmol/L): treatment group 1(110, 50-280); treatment group 2 (90, 50-200)  Number: treatment group 1(19); treatment group 2 (21)  Mean age, range (years): treatment group 1 (47, 26-66); treatment group 2 (40, 18-65)  Sex (M/F): treatment group 1(12/7); treatment group 2 (14/7) |
|  | Interventions | Treatment group 1:  Oral CTX: maximum dosage of l.5 mg/kg/d for 6 mouths  Dipyridamole and sodium warfarin therapy were continued for 2 years Treatment group 2:  Symptomatic treatment only |
|  | Outcomes | Partial or complete remission  Composite endpoint of mortality or ESRD  Withdrawal of treatments |
|  | Methods | Study design: parallel RCT Study duration: before 1979 |
| Coggins  (1979) | Participants | Country: USA  Setting: multicentre  Inclusion criteria: Patients with biopsy-proven IMN and nephrotic syndrome  Characteristics of the patients at baseline: Proteinuria (g/24 h): treatment group 1(9.4 ± 6); treatment group 2 (8.3 ± 4)  SCr (mg/dL): treatment group 1(1.1 ± 0.2); treatment group 2 (1.0 ± 0.2)  Number: treatment group 1(34); treatment group 2 (38)  Mean age, range (years): 39, 16-65  Sex (M/F): treatment group 1(22/12); treatment group 2 (20/18) |
|  | Interventions | Treatment group 1:  Prednisone: Weight 45 to 80 kg: 125 mg qd; Weight < 45 kg: 100 mg qd; Weight > 80 kg: 150 mg qd.  Treatment group 2:  Placebo: identical placebo control tablets (supplied by Upjohn Company) |
|  | Outcomes | Partial or complete remission  Composite endpoint of mortality or ESRD  Withdrawal of treatments |
|  | Methods | Study design: open, parallel RCT  Study duration: before 1994 |
| Ahmed  (1994) | Participants | Country: Bangladesh  Setting: single-center  Inclusion criteria: Patients with biopsy-proven IMN with nephrotic syndrome; SCr < 1.7 mg/dL  Characteristics of the patients at baseline: Proteinuria (g/24 h): treatment group 1 (6.11 ± 1.86); treatment group 2 (7.61 ± 1.99)  Serum albumin: NS  SCr (mg/dL): treatment group 1 (1.35 ± 0.13); treatment group 2 (1.22 ± 0.16)  Number: treatment group 1 (10); treatment group 2 (10)  Mean age ± SD (years): treatment group 1 (32 ± 7); treatment group 2 (38 ± 14)  Sex (M/F): treatment group 1 (8/2); treatment group 2 (8/2) |
|  | Interventions | Treatment group 1:  Methylprednisolone: 1 g/d IV for 3 consecutive days  Prednisolone: 0.5 mg/kg/d for 27 days  Chlorambucil: 0.2 mg/kg/d for 1 month for 3 cycles (6 months)  Treatment group 2:  Prednisolone: 1.0 to 1.5 mg/kg/d for 8 weeks and then in tapering dose and finally withdrawal after 8 weeks |
|  | Outcomes | Partial or complete remission  Infection  Bone marrow suppression  Incidence of hypertension |
|  | Methods | Study design: parallel RCT Study duration: before 1993 |
| Pahari  (1993) | Participants | Country: India  Setting: NS  Inclusion criteria: Patients with biopsy-proven IMN and > 2.0 g/24 h proteinuria  Characteristics of the patients at baseline: Proteinuria (g/24 h): ≥2  Serum albumin: NS  SCr (mg/dL): ≤2  Number: treatment group 1 (36); treatment group 2 (35)  Mean age ± SD (years): treatment group 1 (35 ± 16); treatment group 2 (32 ± 20)  Sex (M/F): treatment group 1 (25/11); treatment group 2 (24/11) |
|  | Interventions | Treatment group 1:  Oral prednisolone: 4 mg/kg/d from 1 to 3 days followed by oral prednisolone 0.5 mg/kg/d from 4 to 30 days (Injection dexamethasone 1 mg/kg/d from 1 to 3 days in cases who are intolerant to high dose oral prednisolone)  Oral CTX: 2 mg/kg/d from 1 to 30 days of next months (oral chlorambucil was used in patients intolerant to oral CTX). The treatment was continued for 1 year. Treatment group 2:  Oral prednisolone: 60 mg/d was given for 12 weeks |
|  | Outcomes | Partial or complete remission  Composite endpoint of mortality or ESRD  Withdrawal of treatments |
|  | Methods | Study design: parallel RCT Study duration: 1974-1980 |
| Tiller  (1981) | Participants | Country: Australia  Setting: multicenter  Inclusion criteria: Patients with biopsy-proven IMN and nephrotic syndrome  Characteristics of the patients at baseline: Proteinuria (g/24 h): treatment group (5.0); control group (4.2)  Serum albumin: NS  SCr: patients with SCr > 350 µmol/L were excluded  Number: treatment group (27); control group (27) |
|  | Interventions | Treatment group 1:  CTX was given at a dosage of l.5 mg/kg/d for 6 mouths  Dipyridamole and sodium warfarin therapy were prescribed  Treatment group 2:  Symptomatic treatment |
|  | Outcomes | Composite endpoint of mortality or ESRD  Withdrawal of treatments  Infection  Bone marrow suppression |
|  | Methods | Study design: parallel RCT Study duration: 1989-1992 |
| Koshikawa (1993) | Participants | Country: Japan  Setting: NS  Inclusion criteria: Patients with biopsy-proven IMN with steroid-resistant nephrotic syndrome  Characteristics of the patients at baseline: Proteinuria (g/24 h): NS  SCr (umol/L): NS  GFR (mL/min): ≥50  Number: treatment group 1(48); Treatment group 2 (41)  Age: > 15 years |
|  | Interventions | Treatment group 1:  Mizoribine: 50 mg 3 times/d after meals for 24 weeks  Treatment group 2:  Placebo |
|  | Outcomes | Partial or complete remission  Withdrawal of treatments |
|  | Methods | Study design: parallel RCT Study duration: 2009-2013 |
| Lei P (2016) | Participants | Country: China  Setting: single-center  Inclusion criteria: All participating patients signed the informed consent. They all received a diagnosis of IMN by renal biopsy and laboratory examination. All of them had persistent proteinuria (> 8 g/d) after observation for at least 1 month and met the diagnostic criteria for nephrotic syndrome.  Characteristics of the patients at baseline: Proteinuria (g/24 h): treatment group 1 (11.7±3.2); treatment group 2 (11.9 ± 1.5); treatment group 3 (11.2 ± 3.7) SCr (umol/L): treatment group 1 (82.4 ± 13.6); treatment group 2 (78.4 ± 13.8) ; treatment group 3 (78.7 ±13.8)  GFR (mL/min): treatment group 1 (87.9±16.5); treatment group 2 (97.3 ±23.0) ; treatment group 3 (95.8 ± 24.9)  Alb (g/L): treatment group 1 (20.5±3.4); treatment group 2 (19.8 ±3.8) ; treatment group 3 (21.9 ± 4.9) Number: treatment group 1 (30); treatment group 2 (30) ; treatment group 3 (30) Mean age ± SD (years):  treatment group 1 (43.9±13.2); treatment group 2 (40.8±13.3) ; treatment group 3 (39.9±14.3) Sex (M/F): treatment group 1 (17/13); treatment group 2 (16/14) ; treatment group 2 (14/16) |
|  | Interventions | Treatment group 1:  Tacrolimus was administered at 0.05 mg/kg/d divided into two doses at intervals of 12 hours initially. The dose was adjusted to achieve a blood trough concentration of 4-8 ng/mL for 6 months and then reduced to 2-4 ng/mL in the subsequent 3 months.  Oral corticosteroid was administered at a dose of 0.5 mg/kg/d Treatment group 2:  CTX was administered by intravenous injection at a the dose of 750 mg/m2 once a month for 6 months, which was then reduced to 750 mg/m2 every 3 months.  Oral corticosteroid was administered at a dose of 1 mg/kg/d  Treatment group 3:  Patients received MMF at 1.5e2.0 g/d in two doses  Oral corticosteroid was administered at a dose of 1 mg/kg/d |
|  | Outcomes | Partial or complete remission  Composite endpoint of mortality or ESRD  Withdrawal of treatments  Infection  Bone marrow suppression  Abnormal liver function  Incidence of DM |
|  | Methods | Study design: parallel RCT Study duration: before 2015 |
| Raja  (2016) | Participants | Country: India  Setting: single-center  Inclusion criteria: Adults (18–60years) with biopsy proved IMN based on light microscopy and immunofluorescence with persistent NS (defined below) in spite of 6 months of treatment with either angiotensin converting enzyme inhibitor (ACEi)/angiotensin receptor blocker (ARBs) or anyone with deep vein thrombosis (DVT)  Characteristics of the patients at baseline: Proteinuria (g/24 h): treatment group 1 (6.76±3.59); treatment group 2 (5.44 ± 2.66) SCr (mg/dL): treatment group 1 (0.90 ± 0.27); treatment group 2 (0.91 ± 0.26)  GFR (mL/min): treatment group 1 (96.72±27.13); treatment group 2 (89.04 ±27.63) Number: treatment group 1 (35); treatment group 2 (35) Mean age ± SD (years):  treatment group 1 (38.66±1.91); treatment group 2 (40.80±10.64) Sex (M/F): treatment group 1 (27/8); treatment group 2 (20/15) |
|  | Interventions | Treatment group 1:  Oral tacrolimus 0.1 mg/kg per day was given in two divided doses for one year to keep trough levels as estimated by micro particle enzyme linked-immunoassay at 5–10 ng/mL in 1st 6 months and 4–8 ng/mL in the next 6 months.  Oral prednisolone was given in a dose of 0.5 mg/kg per day for 6 months and was then tapered and stopped Treatment group 2:  Intravenous methylprednisolone 1 g/day in 100 mL normal saline was administered over 60 min on three consecutive days followed by oral prednisolone 0.5 mg/kg per day for 27 days in the first, third, and fifth month and oral CTX at 2 mg/kg per day in the second, fourth, and sixth month |
|  | Outcomes | Partial or complete remission  Infection  Bone marrow suppression  Incidence of hypertension  Incidence of DM |
|  | Methods | Study design: RCT Study duration: before 2015 |
| Liu SZ  (2015) | Participants | Country: China  Setting: single-center  Inclusion criteria: Patients with biopsy-proven IMN and nephrotic syndrome  Characteristics of the patients at baseline: Proteinuria (g/24 h):NS  SCr (umol/L): NS  GFR (mL/min):NS  Number: treatment group 1 (24); treatment group 2 (24) |
|  | Interventions | Treatment group 1:  glucocorticoid + cyclophosphamide Treatment group 2:  glucocorticoid + leflunomide |
|  | Outcomes | Partial or complete remission |
|  |  |  |
